# Transient Phosphenium and Arsenium Ions versus Stable Stibenium and Bismuthenium Ions

**DOI:** 10.1002/chem.201902520

**Published:** 2019-10-24

**Authors:** Marian Olaru, Daniel Duvinage, Enno Lork, Stefan Mebs, Jens Beckmann

**Affiliations:** ^1^ Institut für Anorganische Chemie und Kristallographie Universität Bremen Leobener Straße 7 28359 Bremen Germany; ^2^ Institut für Experimentalphysik Freie Universität Berlin Arnimallee 14 14195 Berlin Germany

**Keywords:** arsenium ion, fluoride abstraction, Lewis acid, *m*-terphenyl, phosphenium ion

## Abstract

Fluoride abstraction from bis‐*m*‐terphenylelement fluorides (2,6‐Mes_2_C_6_H_3_)_2_EF (E=P, As) generated the highly reactive phosphenium ion [(2,6‐Mes_2_C_6_H_3_)_2_P]^+^ and the arsenium ion [(2,6‐Mes_2_C_6_H_3_)_2_As]^+^, which immediately underwent intramolecular electrophilic substitution and formation of an 1,2,4‐trimethyl‐6‐mesityl‐5‐*m*‐terphenyl‐benzo[*b*]phospholium ion and an 1,2,4‐trimethyl‐6‐mesityl‐5‐*m*‐terphenyl‐benzo[*b*]arsolium ion, respectively. The formation of the latter involved a methyl group migration from the *ortho*‐position of a flanking mesityl group to the *meta*‐position. This reactivity of [(2,6‐Mes_2_C_6_H_3_)_2_E]^+^ (E=P, As) is in sharp contrast to the related stibenium ion [(2,6‐Mes_2_C_6_H_3_)_2_Sb]^+^ and bismuthenium ion [(2,6‐Mes_2_C_6_H_3_)_2_Bi]^+^, which have been recently isolated and fully characterized (*Angew. Chem. Int. Ed*. 2018, **57**, 10080–10084). On the basis of DFT calculations, a mechanism for the rearrangement of the phosphenium and arsenium ions into the phospholium and arsolium ions is proposed, which is not feasible for the stibenium and bismuthenium ions.

## Graphical abstract

**The fluoride abstraction** from the bis(*m*‐terphenyl)element fluorides (P, As) using the Lewis acids EtAlCl_2_ and [Et_3_Si(toluene)]^+^, respectively, provided the transient phosphenium and arsenium ion [(2,6‐Mes_2_C_6_H_3_)_2_As]^+^ which rearrange readily into the protonated 9‐phospha‐ and 9‐arsena‐fluorenes.

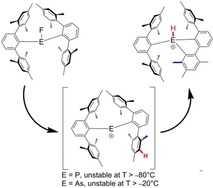

Divalent phosphenium ions, [R_2_P]^+^, and arsenium ions, [R_2_As]^+^, are six‐valence electron species, containing an electron lone pair as well as a vacant *p*‐orbital, which consequently possess Lewis amphoteric properties.^1^ Compared to the neutral isoelectronic group 14 carbene analogues, R_2_E (E=Si, Ge),^2^ the cationic group 15 analogues [R_2_E]^+^ (E=P, As, Sb, Bi) are much stronger electrophiles. In fact, the simplest donor‐free dialkyl‐ and diarylphosphenium ions, [Me_2_P]^+^ and [Ph_2_P]^+^ are predicted to be strong Lewis super acids in the gas phase.^3^ Therefore, the vast majority of phosphenium and arsenium ions known in condensed phase are intra‐ or intermolecularly stabilized by substituents or ligands that compensate the electron deficiency by conjugation with donor atoms possessing electron lone pairs, which dramatically reduces the Lewis acidity and reactivity.^1^ Since the seminal work of Dimroth and Hoffmann, published in 1964,^4^ many of these electron‐rich, donor‐stabilized phosphenium^5^ and arsenium ions^6^ have been reported. The only disputable exception seems to be the bis(ferrocencyl)phosphenium ion [Fc_2_P]^+^, reported by Cowley et al. in 1981, which, however, was never structurally characterized.^7^ The fact that other main group cations containing ferrocenyl groups, such as [FcPh_2_C]^+[8]^ and [Fc(Me)*t*BuSi]^+^,^9^ show significant intramolecular Fe⋅⋅⋅E (E=C, Si) interaction, casts doubt on the claim that [Fc_2_P]^+^ is a donor‐free phosphenium ion and suggests that a similar Fe⋅⋅⋅P interaction might be present.^10^ The preparation of divalent phosphenium and arsenium ions from neutral trivalent precursors involves the abstraction of one substituent and replacement by a weakly coordinating anion. This is exemplified in the reaction of Ph_2_PCl with GaCl_3_ or [Me_3_Si][FAl(OR^F^)_3_] respectively, providing a phosphino‐phosphonium ion [Ph_2_PPPh_2_Cl]A (A=GaCl_4_,^11^ or F[Al(OR^F^)_3_]_2_;^12^ R^F^=C(CF_3_)_3_), which can be regarded as a donor–acceptor complex between the elusive Lewis acid [Ph_2_P]^+^ and Lewis base Ph_2_PCl. These examples raise the question whether bulky substituents are able to prevent the formation of such dinuclear donor–acceptor complexes.

In this work we describe the fluoride abstraction from (2,6‐Mes_2_C_6_H_3_)_2_PF^13^ and (2,6‐Mes_2_C_6_H_3_)_2_AsF that proceeded most likely with the formation of the transient phosphenium ion [(2,6‐Mes_2_C_6_H_3_)_2_P]^+^ [**1 a**]^+^ and the arsenium ion [(2,6‐Mes_2_C_6_H_3_)_2_As]^+^ [**1 b**]^+^, whereby two bulky *m*‐terphenyl substituents prevent the formation of dinuclear donor–acceptor complexes. Using a similar strategy, we have been recently able to isolate the heavier stibenium ion [(2,6‐Mes_2_C_6_H_3_)_2_Sb]^+^ [**1 c**]^+^ and the bismuthenium ion [(2,6‐Mes_2_C_6_H_3_)_2_Bi]^+^ [**1 d**]^+^ (Scheme 1).^14^

**Scheme 1:**
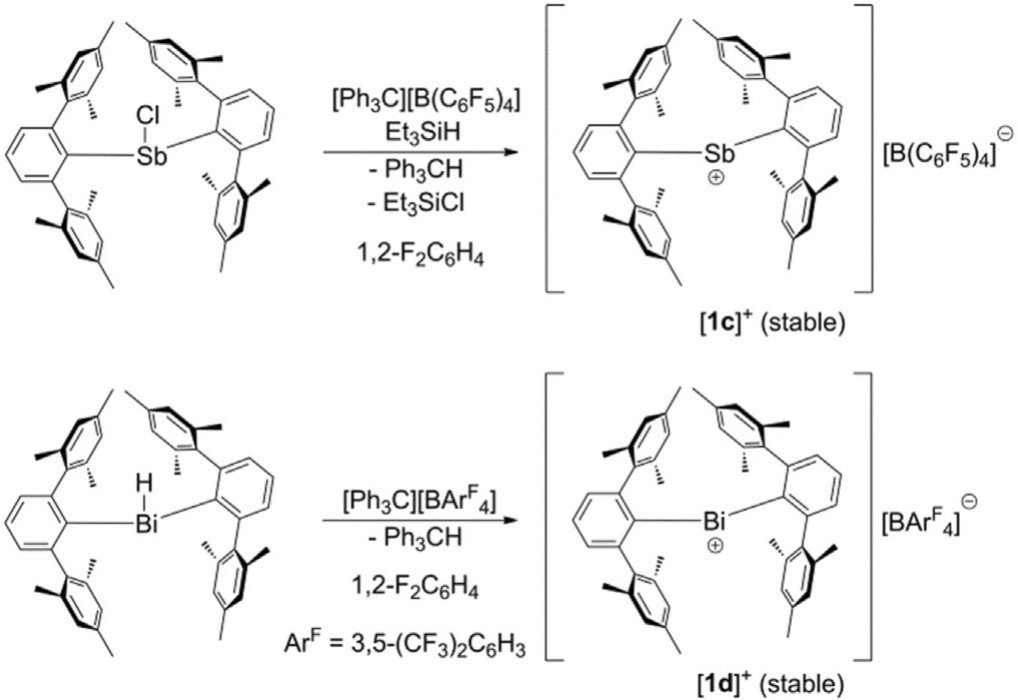
Synthesis of the stibenium ion [**1 c**]^+^ and the bismuthenium ion [**1 d**]^+^.

Unlike the indefinitely stable [**1 c**]^+^ and [**1 d**]^+^, the transient [**1 a**]^+^ and [**1 b**]^+^, immediately undergo intramolecular electrophilic substitution and formation of the 1,2,4‐trimethyl‐6‐mesityl‐5‐*m*‐terphenyl‐benzo[*b*]phospholium ion, [**2 a**]^+^, and the 1,2,4‐trimethyl‐6‐mesityl‐5‐*m*‐terphenyl‐benzo[*b*]arsolium ion, [**2 b**]^+^, respectively. The formation of these protonated 9‐phospha‐ and 9‐arsena‐fluorenes [**2 a**]^+^ and [**2 b**]^+^ involved a 1,2‐methyl shift in one of the flanking mesityl groups of the *m*‐terphenyl substituent, for which a mechanism is proposed on the basis of DFT calculations.

The starting materials (2,6‐Mes_2_C_6_H_3_)_2_EF (E=P, As) were prepared in a one pot reaction from 2,6‐Mes_2_C_6_H_3_Li, ECl_3_ and CsF, as recently communicated already for the phosphorus compound (Figure 1).^14^ Unlike many other examples of this compound class, (2,6‐Mes_2_C_6_H_3_)_2_EF (E=P, As) show no sign for spontaneous redistribution.^14^, ^15^ In solution, they are characterized by ^19^F NMR chemical shifts (CD_2_Cl_2_) of *δ*=−197.6 (E=P) and −209.7 ppm (E=As). Comparison of the molecular structure reveals that the C−P−C bond angle (106.1(1)°) is slightly smaller than the C−As−C bond angle (112.8(1)°). The attempted fluoride abstraction of (2,6‐Mes_2_C_6_H_3_)_2_PF with the strong electrophile [Et_3_Si(toluene)][B(C_6_F_5_)_4_] gave rise to the formation of the donor acceptor complex [R_2_(F)PSiEt_3_][B(C_6_F_5_)_4_] only.^14^ Interestingly, fluoride abstraction was achieved using an excess of the weaker electrophiles EtAlCl_2_ or AlCl_3_ in CH_2_Cl_2_ and/or heptane. Due to the higher solubility of EtAlCl_2_ in most organic solvents the results were better than with AlCl_3_.

**Figure 1:**
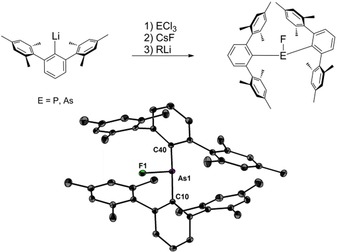
Synthesis of the bis(*m*‐terphenyl)element fluorides. Molecular structure of (2,6‐Mes_2_C_6_H_3_)_2_AsF showing 50 % probability ellipsoids and the essential atom numbering Scheme. Selected bond parameters [Å, °]: As1−F1 1.764(1), As1−C10 1.992(1), As1−C40 1.993(1), C10‐As1‐C40 112.8(1).

Thus, the reaction of (2,6‐Mes_2_C_6_H_3_)_2_PF with EtAlCl_3_ at −90 °C provided a dark solution, reminiscent of the color of solutions containing the stibenium ion [(2,6‐Mes_2_C_6_H_3_)_2_Sb]^+^, [**1 c**]^+^ or the bismuthenium ion [(2,6‐Mes_2_C_6_H_3_)_2_Bi]^+^, [**1 d**]^+^.^14^ Above −80 °C, the dark color rapidly faded away to give a yellow solution, which contained the 1,2,4‐trimethyl‐6‐mesityl‐5‐*m*‐terphenyl‐benzo[*b*]phospholium ion, [**2 a**]^+^ as the major product (Figure 2). In solution, this protonated 9‐phospha‐fluorene [**2 a**]^+^ is characterized by a ^31^P NMR chemical shift (CD_2_Cl_2_) of *δ*=−15.0 (E=P) and a ^1^ *J*(^1^H‐^31^P) coupling of 501 Hz. Under inert conditions, solution of [**2 a**]^+^ are stable for at least several days. Unfortunately, all attempts to isolate or crystallize [**2 a**]^+^ as aluminate salt from these solutions failed. We therefore added triethylamine to induce deprotonation of [**2 a**]^+^, which provided the related neutral phosphole **3 a** as crystalline solid (Figure 2). In solution, 9‐phospha‐fluorene **3 a** gives rise to a ^31^P NMR chemical shift (CD_2_Cl_2_) of *δ*=−25.2 ppm. Re‐protonation of **3 a** using HCl/Na[BAr^F^_4_] eventually afforded [**2 a**]^+^ as crystalline [BAr^F^_4_]^−^ salt (Figure 2, Ar^F^ = 3,5‐(CF_3_)_2_C_6_H_3_). We note in passing that [**2 a**]^+^ does not react further with excess EtAlCl_2_ unlike **3 a**, which readily undergoes oxidative addition^16^ with EtAlCl_2_ in CH_2_Cl_2_ to give 5‐(chloromethyl)‐5‐*m*‐terphenyl‐6‐mesityl‐1,2,4‐trimethyl‐benzo[*b*]‐phospholium salt [**4**][AlCl_4_] as major species (see the Supporting Information). Inspection of the molecular structures of [**2 a**]^+^ and **3 a** revealed that the initially formed phosphenium ion had undergone an intramolecular electrophilic attack of a flanking mesityl groups at one of the *m*‐terphenyl substituents. This attack occurred in *ortho*‐position of the mesityl group and proceeded with cleavage of the methyl group, which migrated to the *meta*‐position. Unlike (2,6‐Mes_2_C_6_H_3_)_2_PF, the fluoride abstraction of (2,6‐Mes_2_C_6_H_3_)_2_AsF was achieved with [Et_3_Si(toluene)][B(C_6_F_5_)_4_] at −40 °C to give initially the arsenium cation [**1 b**]^+^, which, however, rearranged into the 1,2,4‐trimethyl‐6‐mesityl‐5‐*m*‐terphenyl‐benzo[*b*]arsolium ion, [**2 b**]^+^ above −20 °C (Figure 1). NMR inspection showed that solutions of [**2 b**]^+^ are unstable and give multiple products after a short period of time. We therefore also added triethylamine to induce deprotonation of [**2 b**]^+^. In this way, the related neutral arsole **3 b** was obtained as crystalline solid (Figure 2). Similar as for [**2 a**]^+^ and **3 a**, the molecular structure of **3 b** revealed that an 1,2‐methyl shift in one of the mesityl groups had taken place (Figure 2). We note that Wehmschulte et al. had previously observed the formation of 9‐phospha‐ and 9‐arsafluorenes from *m*‐terphenyldichlorophosphines and ‐arsenes; however, these involved no methyl group migration.^17^

**Figure 2:**
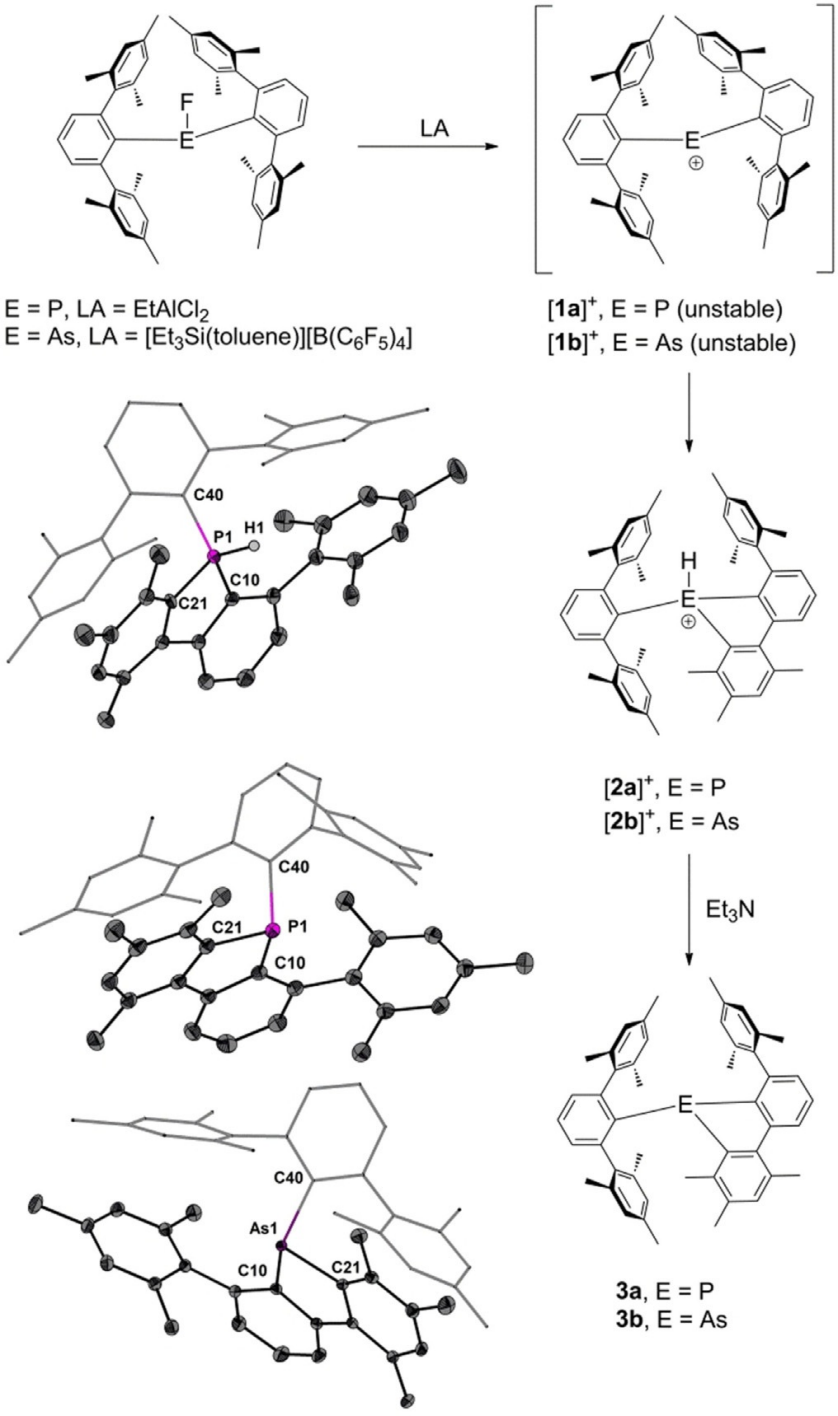
Fluoride abstraction of bis(*m*‐terphenyl)element fluorides. Molecular structures of [**2 a**]^+^, **3 a**, and **3 b** showing 50 % probability ellipsoids and the essential atom numbering Scheme. Selected bond parameters [Å, °] of [**2 a**]^+^: P1−H1 1.31(3), P1−C10 1.793(2), P1−C21 1.788(2), P1−C40 1.809(2), C10‐P1‐C40 123.4(1), C21‐P1‐C10 94.6(1), C21‐P1‐C40 110.9(1). Selected bond parameters [Å, °] of **3 a**: P1−C10 1.838(2), P1−C21 1.832(2), P1−C40 1.871(2), C10‐P1‐C21 89.48(7), C10‐P1‐C40 104.51(6), C21‐P1‐C40 107.26(6). Selected bond parameters [Å, °] of **3 b**: As1−C10 1.957(1), As1−C21 1.954(1), As1−C40 2.002(1), C10‐As1‐C21 85.68(5), C10‐As1‐C40 107.76(5), C21‐As1‐C40 99.43(5).

The fluoride abstraction from (2,6‐Mes_2_C_6_H_3_)_2_PF with [Et_3_Si]^+^ was impaired by the exothermic formation (Δ*E*=−241.0 kJ mol^−1^) of the stable donor–acceptor complex [(2,6‐Mes_2_C_6_H_3_)_2_(F)PSiEt_3_]^+^.^13^, ^18^ The hypothetical elimination of Et_3_SiF and formation of [(2,6‐Mes_2_C_6_H_3_)_2_P]^+^ [**1 a**]^+^ was found to be endothermic (Δ*E*=+40.1 kJ mol^−1^).^13^, ^18^ In comparison, the fluoride abstraction from (2,6‐Mes_2_C_6_H_3_)_2_AsF with [Et_3_Si]^+^ might also form a thermodynamically donor–acceptor complex [(2,6‐Mes_2_C_6_H_3_)_2_(F)AsSiEt_3_]^+^ (Δ*E*=−128.3 kJ mol^−1^); however, the competing elimination into Et_3_SiF and formation of [(2,6‐Mes_2_C_6_H_3_)_2_As]^+^ [**1 b**]^+^ was calculated to be thermodynamically more feasible (Δ*E*=−133.0 kJ mol^−1^), which is fully consistent with the experimental observations.^13^, ^18^ With the substantially weaker electrophile EtAlCl_2_, both the complex formation (2,6‐Mes_2_C_6_H_3_)_2_P(F)AlEtCl_2_ (Δ*E*=33.08 kJ mol^−1^) and the fluoride abstraction to give [(2,6‐Mes_2_C_6_H_3_)_2_P]^+^ [**1 a**]^+^ and [EtAlCl_2_F]^−^ (Δ*E*=210.9 kJ mol^−1^) are predicted to be endothermic. We therefore hypothesized that the active electrophile might have been [EtAlCl]^+^ or [AlCl_2_]^+^, which were formed from EtAlCl_2_ through autoionization. Notably, the autoionization of the AlCl_3_ in donor solvents to give solvated [AlCl_2_]^+^ and [AlCl_4_]^−^ is well established.^19^ The complex formation between (2,6‐Mes_2_C_6_H_3_)_2_PF and [EtAlCl]^+^ or [AlCl_2_]^+^ was indeed calculated to be exothermic (Δ*E*=−343.4 and −448.7 kJ mol^−1^, respectively); however, the fluoride abstraction to give [(2,6‐Mes_2_C_6_H_3_)_2_P]^+^ [**1 a**]^+^, EtAlClF (Δ*E*=44.2 kJ mol^−1^) and AlCl_2_F (Δ*E*=51.5 kJ mol^−1^), respectively, was still calculated to be endothermic, which does not verify our hypothesis conclusively.

To this end, it remains unclear as to why the weak Lewis acid EtAlCl_2_ is able to abstract fluoride from (2,6‐Mes_2_C_6_H_3_)_2_PF.

In an effort to understand why the phosphenium ion [(2,6‐Mes_2_C_6_H_3_)_2_P]^+^ [**1 a**]^+^ and the arsenium ion [(2,6‐Mes_2_C_6_H_3_)_2_As]^+^ [**1 b**]^+^ undergo rearrangement into the phospholium and arsolium ions [**2 a**]^+^ and [**2 b**]^+^, whereas the stibenium ion [(2,6‐Mes_2_C_6_H_3_)_2_Sb]^+^ [**1 c**]^+^ and bismuthenium ion [(2,6‐Mes_2_C_6_H_3_)_2_Bi]^+^ [**1 d**]^+^ are stable, we calculated a conceivable mechanism for the 1,2‐methyl shift for all heteroelements (Scheme 2). The energy of the pnictogenium ions [**1 a**]^+^–[**1 d**]^+^ was arbitrarily set to 0 kJ mol^−1^. Compared to these references, the conversion is strongly exothermic for [**2 a**]^+^ (−209.6 kJ mol^−1^) and [**2 b**]^+^ (−119.0 kJ mol^−1^), weakly exothermic for **2 c**]^+^ (−23.4 kJ mol^−1^) and endothermic for [**2 d**]^+^ (92.3 kJ mol^−1^). The intramolecular electrophilic attack of [**1 a**]^+^–[**1 d**]^+^ gives rise to the formation of arenium ions [**5 a**]^+^–[**5 d**]^+^, followed by the 1,2‐methyl shift and the formation of other arenium ions [**6 a**]^+^–[**6 d**]^+^ and a subsequent proton transfer to give the protonated 9‐pnictogena‐fluorene ions [**2 a**]^+^–[**2 d**]^+^ (Scheme 2). The formation of both types of arenium ions [**5 a**]^+^–[**5 d**]^+^ is exothermic for phosphorus and arsenic, but only very weakly exothermic or endothermic for antimony and bismuth (Scheme 2). The highest transition state energies were found for the migration of the methyl groups from the *ortho*‐positions to the *meta*‐positions. Overall, the energy pathways are consistent with the observed rearrangement of the phosphenium ion [(2,6‐Mes_2_C_6_H_3_)_2_P]^+^ [**1 a**]^+^ and the arsenium ion [(2,6‐Mes_2_C_6_H_3_)_2_As]^+^ [**1 b**]^+^ into the protonated 9‐phospha‐ and 9‐arsena‐fluorenes [**2 a**]^+^ and [**2 b**]^+^ and the observed stability of the stibenium ion [(2,6‐Mes_2_C_6_H_3_)_2_Sb]^+^, [**1 c**]^+^ or the bismuthenium ion [(2,6‐Mes_2_C_6_H_3_)_2_Bi]^+^, [**1 d**]^+^.^14^

**Scheme 2:**
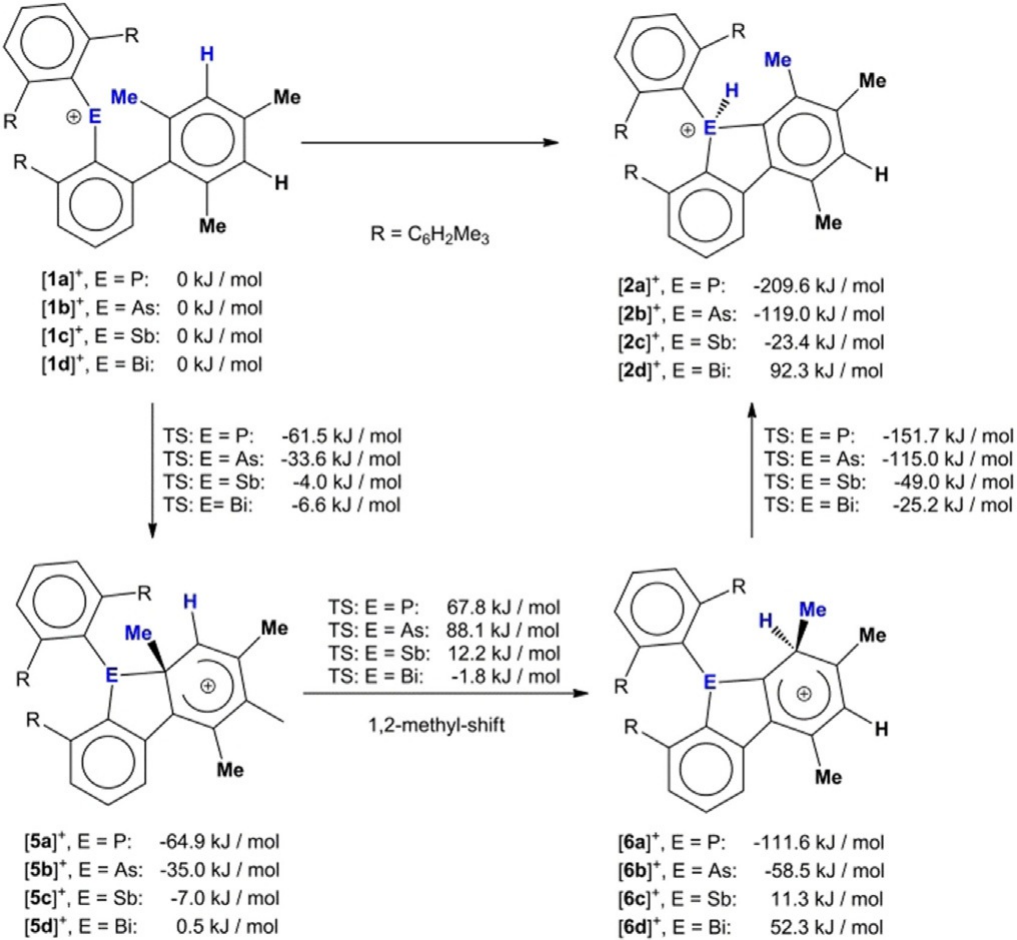
Mechanism of the rearrangement from pnictogenium ions [**1 a**]^+^–[**1 d**]^+^ to the protonated 9‐pnictogena‐fluorene ions [**2 a**]^+^–[**2 d**]^+^.

The fluoride abstraction from the bis(*m*‐terphenyl)element fluorides (2,6‐Mes_2_C_6_H_3_)_2_EF (E=P, As) using the Lewis acids EtAlCl_2_ and [Et_3_Si(toluene)]^+^, respectively, provided the transient phosphenium ion [(2,6‐Mes_2_C_6_H_3_)_2_P]^+^ [**1 a**]^+^ and the arsenium ion [(2,6‐Mes_2_C_6_H_3_)_2_As]^+^ [**1 b**]^+^, which rearrange into protonated 9‐phospha‐ and 9‐arsena‐fluorenes [**2 a**]^+^ and [**2 b**]^+^. The mechanism of the rearrangement involves an intramolecular electrophilic attack, a methyl group migration in one of the flanking mesityl groups and proton transfer to phosphorus and arsenic. This mechanism suggests to avoid reactive hydrogen atoms in the *m*‐terphenyl substituents, for example, by using permethylated phenyl groups in the flanking positions, which is now being investigated in our laboratory. For the stable stibenium ion [(2,6‐Mes_2_C_6_H_3_)_2_Sb]^+^, [**1 c**]^+^ and the bismuthenium ion [(2,6‐Mes_2_C_6_H_3_)_2_Bi]^+^, [**1 d**]^+[14]^ the same mechanism is not feasible on thermodynamic grounds.

## Conflict of interest

The authors declare no conflict of interest.

## Supporting information

As a service to our authors and readers, this journal provides supporting information supplied by the authors. Such materials are peer reviewed and may be re‐organized for online delivery, but are not copy‐edited or typeset. Technical support issues arising from supporting information (other than missing files) should be addressed to the authors.

Supplementary
