# Supplementary material for: Transient Phosphenium and Arsenium Ions versus Stable Stibenium and Bismuthenium Ions
Source: Chemistry. 2019 Oct 24;25(65):14758–61. doi: 10.1002/chem.201902520 (PMC6900177; doi:10.1002/chem.201902520)
Supplement: Supplementary file 1 — Supplementary [file CHEM-25-14758-s001.pdf]

CHEMISTRY
A European Journal

Supporting Information

Transient Phosphenium and Arsenium Ions versus Stable Stibenium and Bismuthenium Ions

Marian Olaru,[a] Daniel Duvinage,[a] Enno Lork,[a] Stefan Mebs,*[b] and Jens Beckmann*[a]

chem_201902520_sm_miscellaneous_information.pdf

Table of Contents

Experimental procedures ... 2

General information ... 2

Synthesis and characterization of (2,6-$Mes_2C_6H_3)_2AsF$ ... 3

Synthesis and characterization of **3a** ... 7

Synthesis and characterization of **3b** ... 20

Synthesis and characterization of [**2a**][BAr^F_4] ... 25

X-Ray diffraction studies ... 32

Computational data ... 35

References ... 36

Experimental procedures

General information

Unless otherwise stated, all reactions and manipulations were performed under inert atmosphere (argon) using anhydrous solvents. Reagents used in this work including PCl_3 and $AsCl_3$ were obtained commercially and were used as received. CsF was dried at 140 °C under reduced pressure. The reagents 2,6-$Mes_2C_6H_3Li$ (Mes = 2,4,6-$Me_3C_6H_2$),[S1] Na[$BAr^F{}_4$],[S2] Li[$B(C_6F_5)_4$][S3] [Ar^F = 3,5-$(F_3C)_2C_6H_3$], [Ph_3C][$B(C_6F_5)_4$][S4,S5] and [Et_3Si(toluene)][$B(C_6F_5)_4$][S6] were prepared following the published procedures. The synthesis of (2,6-$Mes_2C_6H_3)_2PF$ was reported recently.[9] Anhydrous dichloromethane, hexane, tetrahydrofuran and toluene were collected from an SPS800 mBraun solvent purification system and stored over 4 Å molecular sieves. 1,2-Difluorobenzene was degassed and dried under argon over 4 Å molecular sieves. Et_2O was dried by refluxing it over Na/benzophenone under argon atmosphere. Deuterated solvents were degassed and dried over 4 Å molecular sieves under argon.

Unless otherwise noted, NMR spectra were recorded at room temperature on a Bruker Avance 360 and Avance 600 MHz spectrometers. ^1H, $^{13}C\{^1H\}$, $^{11}B\{^1H\}$, ^{31}P and ^{19}F NMR spectra are reported on the δ scale (ppm) and are referenced against $SiMe_4$, $BF_3{\cdot}Et_2O$ (15% in $CDCl_3$), H_3PO_4 (85% in water) and $CFCl_3$, respectively. ^1H and $^{13}C\{^1H\}$ chemical shifts are reported relative to the residual peak of the solvent ($CDHCl_2$ 5.32 ppm for CD_2Cl_2) in the ^1H NMR spectra, and to the peak of the deuterated solvent (CD_2Cl_2 53.84 ppm) in the $^{13}C\{^1H\}$ NMR spectra.[8] The assignment of the ^1H and $^{13}C\{^1H\}$ resonance signals was made in accordance with the COSY, HSQC and HMBC spectra. The labelling schemes are attached to the ^1H and ^{13}C spectra; in the case of **3a**, **3b**, and [**2a**]$^+$ the NMR and the crystal structure labelling schemes are identical.

The ESI HRMS spectra were measured on a Bruker Impact II spectrometer. Acetonitrile or dichloromethane/acetonitrile solutions ($c = 1{\cdot}10^{-5}$ mol·L^{-1}) were injected directly into the spectrometer at a flow rate of 3 μL·min^{-1}. Nitrogen was used both as a drying gas and for nebulization with flow rates of approximately 5 L·min^{-1} and a pressure of 5 psi. Pressure in the mass analyzer region was usually about $1{\cdot}10^{-5}$ mbar. Spectra were collected for 1 min and averaged. The nozzle-skimmer voltage was adjusted individually for each measurement.

Synthesis and characterization of (2,6-$Mes_2C_6H_3)_2AsF$

A solution of 2,6-$Mes_2C_6H_3Li$ (4.00 g, 12.5 mmol) in Et_2O (50 mL) was added over a solution of $AsCl_3$ (2.50 g, 13.9 mmol) in Et_2O (50 mL) at room temperature over the course of 20 minutes. The reaction mixture was stirred for 2 hours at room temperature and the solvent was removed under reduced pressure. To the crude mixture CsF (6.30 g, 41.3 mmol) and THF (60 mL) were added. The reaction mixture was stirred for 18 h and the solvent was removed under vacuum. To the residue additional 2,6-$Mes_2C_6H_3Li$ (4.00 g, 12.5 mmol) and hexane (40 mL) were added. The mixture was stirred for 18 hours and the reaction was monitored by ^{19}F NMR. The solvent was removed under reduced pressure and the crude product was extracted with CH_2Cl_2 (60 mL). The solvent was removed under vacuum and the remaining beige solid was washed with acetonitrile (3×50 mL) and cold (0 °C) acetone (1×10 mL) and dried at 80 °C ($5\cdot10^{-2}$ mbar) to yield (2,6-$Mes_2C_6H_3)_2AsF$ as a white solid (5.10 g, 56%).

^1H NMR (360 MHz, CD_2Cl_2): δ = 7.25 (t, $^3J(^1H–^1H)$ = 8 Hz, 2H, H4), 6.73 (m, 6H, H3, H7, H9), 2.30 (s, 12H, H13), 1.70 (s, 12H, H12), 1.66 (s, 12H, H11). **$^{13}C\{^1H\}$ NMR (91 MHz, CD_2Cl_2)**: δ = 146.54 (s, C2), 146.05 (d, $^2J(^{13}C–^{19}F)$ = 12 Hz, C1), 139.68 (s, C5), 137.43 (s, C10), 136.94 (s, C8), 136.68 (s, C6), 131.95 (s, C3), 129.73 (s, C4), 128.78 (s, C9), 128.64 (s, C7), 22.28 (s, C12), 22.21 (s, C11), 21.28 (s, C13). **$^{19}F\{^1H\}$ NMR (360 MHz, CD_2Cl_2)**: δ = −209.66 (s). **HRMS ESI (m/z):** $[M+Na]^+$ calculated. for $C_{48}H_{50}AsFNa$, 743.30047; found, 743.30012.

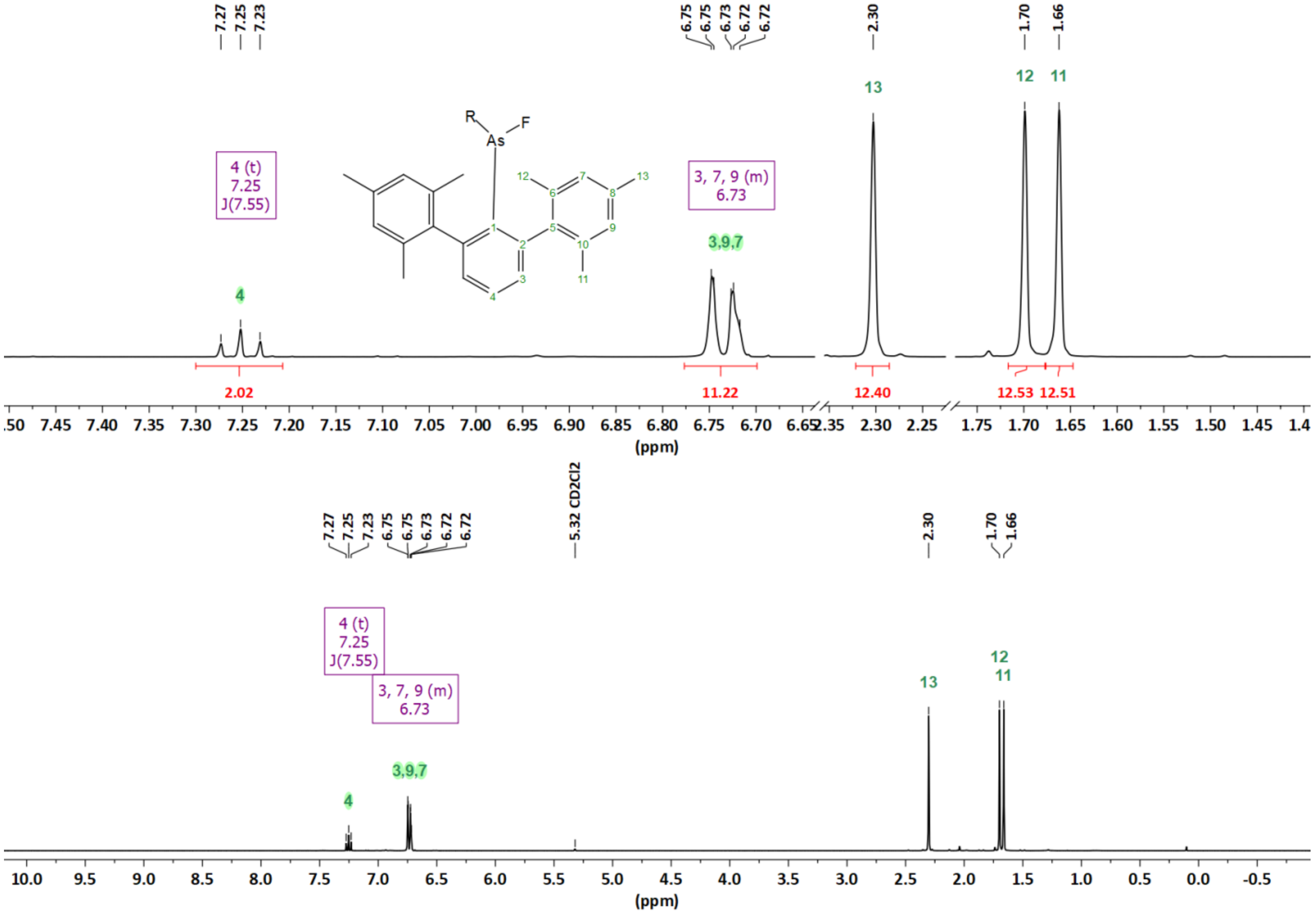

Figure S1. ^{1}H NMR (CD_2Cl_2, 360 MHz) spectrum of $(2,6\text{-}Mes_2C_6H_3)_2AsF$

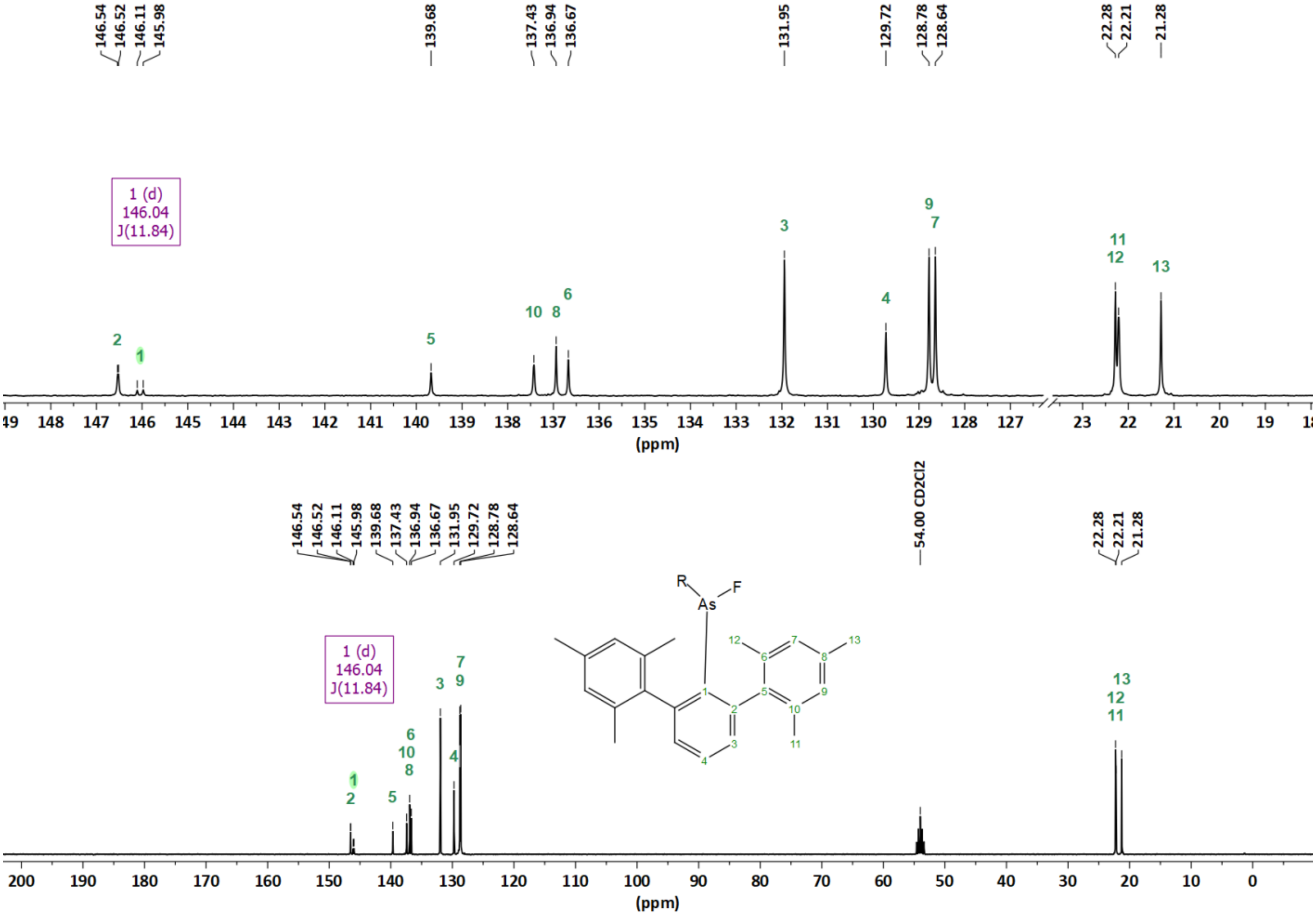

Figure S2. $^{13}C\{^1H\}$ NMR (CD_2Cl_2, 91 MHz) spectrum of (2,6-$Mes_2C_6H_3$)$_2$AsF.

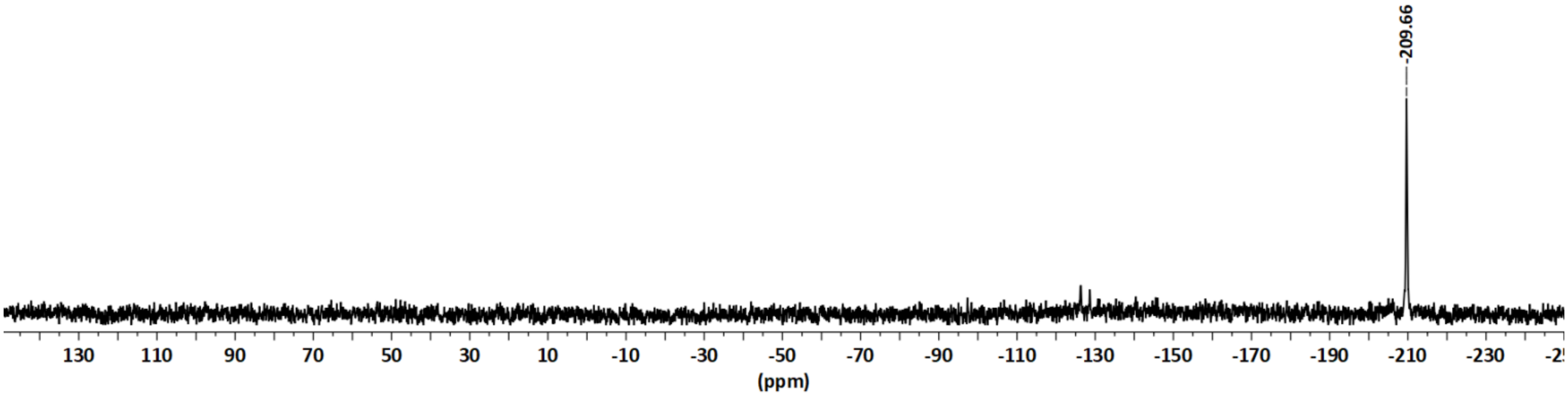

Figure S3. $^{19}F\{^{1}H\}$ NMR (CD_2Cl_2, 188 MHz) spectrum of (2,6-$Mes_2C_6H_3)_2AsF$.

Synthesis and characterization of 3a

To a suspension of (2,6-$Mes_2C_6H_3)_2PF$ (330 mg, 0.49 mmol) in hexane (10 mL) a solution of $EtAlCl_2$ in heptane (0.9 M, 2.6 mL, 2.34 mmol) was added at room temperature. The reaction mixture was stirred at room temperature for 30 minutes. The solution was separated and the residue washed with 10 mL of hexane. Hexane (5 mL), toluene (5 mL) and Et_3N (0.3 mL, 2.15 mmol) were added and after 15 minutes the mixture was filtered through a pad (2 cm) of aluminium oxide. The reaction flask and the pad were washed with a 1:1 mixture of hexane (20 mL) and toluene (20 mL). All volatiles were removed by rotary evaporation. The colourless oil was triturated with a small amount of MeOH and the solid dried under reduced pressure. A mixture of (2,6-$Mes_2C_6H_3)_2PF$ (variable amount) and the title product is obtained (220 mg). The separation of (2,6-$Mes_2C_6H_3)_2PF$ is rather difficult and can be done by careful crystallization of (2,6-$Mes_2C_6H_3)_2PF$ (over several days at −40 °C) from a solution of the mixture in the minimum volume (6-7 mL) of boiling heptane. Alternatively, the mixture can be reacted again with $EtAlCl_2$ in order to completely consume (2,6-$Mes_2C_6H_3)_2PF$. For example: to a mixture of (2,6-$Mes_2C_6H_3)_2PF$ and **3a** (140 mg, 1:10 molar ratio) in hexane[a] (7 mL) was added a solution of $EtAlCl_2$ (0.9 M, 1 mL, 0.90 mmol). The suspension was stirred for 30 minutes then Et_3N (0.3 mL, 2.15 mmol) and toluene (7 mL) were added and the mixture was filtered through a pad (2 cm) of aluminium oxide. The reaction flask and the pad were washed with a 1:1 mixture of hexane (20 mL) and toluene (20 mL). All volatiles were removed by rotary evaporation. The colourless oil was triturated with a small amount of MeOH and the solid dried under reduced pressure to obtain **3a** (130 mg, 94%) as a white solid.

3a:

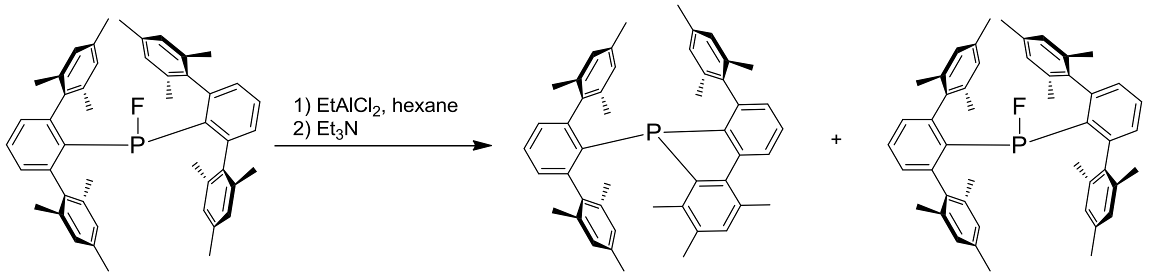

3a

^{1}H NMR (600 MHz, CD_2Cl_2): δ =7.68 (d, $^3J(^1H–^1H)$ = 8 Hz, 1H, H12), 7.38 (t, $^3J(^1H–^1H)$ = 8 Hz, 1H, H13)m, 7.31 (t, $^3J(^1H–^1H)$ = 8 Hz, 1H, H43), 6.91 (dd, $^3J(^1H–^1H)$ = 8 Hz, $^4J(^1H–^1H)$ = 4 Hz,

[a] If the reaction is performed in CH_2Cl_2 a mixture of 5-(chloromethyl)-5-*m*-terphenyl-6-mesityl-1,3,4-trimethyl-benzo[b]phospholium salt [**4**][$AlCl_4$] (major species) and [**2a**][A] (minor species, A = $EtAlCl_3$ or $AlCl_4$). [**4**][$AlCl_4$] can be isolated by recrystallization of the crude mixture of products from $CHCl_3$ and Et_2O. Although for [**4**]$^+$ we expected [$EtAlCl_3$]$^-$ as an counter ion, neither the NMR of the isolated product nor the structure determined (by single crystal X-ray diffraction) after recrystallization of the sample indicated any [$EtAlCl_3$]$^-$, most likely due to redistribution reactions.

1H, H44), 6.84 (s, 1H, H6), 6.80 (s, 1H, H24), 6.78 (s, 2H, H32, H34), 6.72 (s, 1H, H62), 6.64 (m, 2H, H14, H42), 6.41 (s, 1H, H54), 6.04 (s, 1H, H52), 2.39 (s, 3H, H28), 2.33 (s, 3H, H37), 2.31 (s, 3H, H67), 2.20 (s, 3H, H57), 2.18 (s, 3H, H27), 2.06 (s, 3H, H26), 1.82 (s, 3H, H68), 1.76 (s, 3H, H36), 1.50 (s, 3H, H38), 1.38 (s, 3H, H58), 1.26 (s, 3H, H66), 1.17 (s, 3H, H56). **^{13}C{^{1}H} NMR (151 MHz, CD_2Cl_2)**: δ = 152.77 (d, $^{2}J(^{13}C–^{31}P)$ = 42 Hz, C45), 150.57 (s, C11), 149.29 (d, $^{2}J(^{13}C–^{31}P)$ = 7 Hz, C41), 145.67 (d, $^{2}J(^{13}C–^{31}P)$ = 22 Hz, C15), 142.01 (s, C21), 240.88 (s, C20, C30), 140.76 (d, $^{1}J(^{13}C–^{31}P)$ = 6 Hz, C10), 140.44 (d, $^{3}J(^{13}C–^{31}P)$ = 8 Hz, C60), 137.80 (s, C65), 137.50 (d, $^{3}J(^{13}C–^{31}P)$ = 10 Hz, C50), 137.46 (s, C33), 137.30 (d, C35, C61), 137.01 (s, C51), 136.86 (s, C63), 136.74 (s, C55), 136.67 (s, C31), 136.36 (d, $^{2}J(^{13}C–^{31}P)$ = 21 Hz, C22), 135.67 (s, C53), 134.70 (s, C23), 134.66 (s, C24), 132.93 (d, $^{1}J(^{13}C–^{31}P)$ = 35 Hz, C40), 131.82 (s, C25), 131.60 (s, C42), 130.46 (d, $^{3}J(^{13}C–^{31}P)$ = 9 Hz, C44), 130.19 (s, C43), 129.62 (s, C13), 129.51 (s, C32), 129.02 (d, $^{3}J(^{13}C–^{31}P)$ = 5 Hz, C14), 128.94 (s, C34), 128.22 (s, C54), 128.20 (s, C64), 127.75 (s, C62), 127.63 (s, C52), 124.21 (s, C12), 23.39 (s, C28), 22.21 (s, C68), 21.29 (s, C37, C57, C67), 21.18 (s, C58), 20.89 (d, $^{5}J(^{13}C–^{31}P)$ = 8 Hz, C38), 20.59 (d, $^{3}J(^{13}C–^{31}P)$ = 16 Hz, C26), 20.17 (d, $^{5}J(^{13}C–^{31}P)$ = 6 Hz, C66), 19.80 (d, $^{2}J(^{13}C–^{31}P)$ = 11 Hz, C27). **^{31}P NMR (CD_2Cl_2, 243 MHz)**: δ = −25.21. **HRMS ESI (m/z):** $[M+Na]^+$ calculated. for $C_{48}H_{49}PNa$, 679.34604; found, 679.34641.

[**4**][$AlCl_4$]:

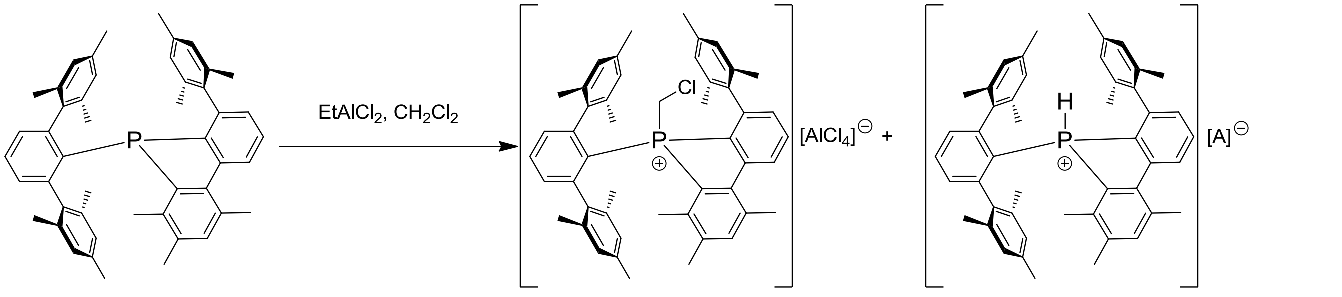

^{1}H NMR (600 MHz, CD_2Cl_2): δ = 7.89–7.84 (m, 3H, H12, H143, H13), 7.35–7.33 (m, 1H, H42), 7.20 (s, 1H, H24), 7.19–7.17 (m, 1H, H44), 7.01 (s, 1H, H52), 6.98 (s, 1H, H32), 6.93–6.91 (m, 1H, H14), 6.89 (s, 1H, H54), 6.82 (s, 1H, H34), 6.60 (s, 1H, H64), 6.12 (s, 1H, H62), 3.78 (dd, $^{2}J(^{1}H–^{31}P)$ = 14 Hz, $^{2}J(^{1}H–^{1}H)$ = 6 Hz, 1H, H1), 3.11 (dd, $^{2}J(^{1}H–^{31}P)$ = 14 Hz, $^{2}J(^{1}H–^{1}H)$ = 5 Hz, 1H, H1), 2.46 (s, 3H, H28), 2.34 (two singlets partially overlapped, 6H, H37, H57), 2.30 (s, 6H, H26, H27), 2.19 (s, 3H, H67), 1.81 (s, 3H, H56), 1.69 (3H, H36), 1.47 (s, 3H, H38), 1.39 (two singlets partially overlapped, 6H, H66, H68), 1.26 (3H, H58). **^{13}C{^{1}H} NMR (151 MHz, CD_2Cl_2)**: δ = 151.24 (d, $^{2}J(^{13}C–^{31}P)$ = 11 Hz, C41), 150.88 (d, $^{2}J(^{13}C–^{31}P)$ = 20 Hz, C11), 150.63 (d, $^{2}J(^{13}C–^{31}P)$ = 11 Hz, C45), 148.35 (d, $^{2}J(^{13}C–^{31}P)$ = 10 Hz, C15), 141.64 (d, $^{4}J(^{13}C–^{31}P)$ = 2 Hz, C24), 141.03 (s, C53), 140.76 (d, $^{2}J(^{13}C–^{31}P)$ = 18 Hz, C22), 140.29 (s, C33),

139.28 (d, $^{3}J(^{13}C–^{31}P)$ = 12 Hz, C23), 139.19 (s, C63), 138.84 (s, C31), 138.28 (s, C51), 137.43 (d, $^{3}J(^{13}C–^{31}P)$ = 7 Hz, C20), 137.88 (s, C61), 137.52 (s, C65), 137.43 (d, $^{4}J(^{13}C–^{31}P)$ = 2 Hz, C13), 137.22 (s, C35), 136.38 (d, $^{4}J(^{13}C–^{31}P)$ = 3 Hz, C43), 136.36 (s, C55), 135.74 (d, $^{3}J(^{13}C–^{31}P)$ = 3 Hz, C50), 135.14 (d, $^{3}J(^{13}C–^{31}P)$ = 11 Hz, C25), 135.02 (d, $^{3}J(^{13}C–^{31}P)$ = 3 Hz, C30), 134.41 (d, $^{3}J(^{13}C–^{31}P)$ = 12 Hz, C44), 134.25 (d, $^{3}J(^{13}C–^{31}P)$ = 4 Hz, C60), 134.18 (d, $^{3}J(^{13}C–^{31}P)$ = 12 Hz, C42), 134.00 (d, $^{3}J(^{13}C–^{31}P)$ = 12 Hz, C14), 130.89 (s, C34), 130.00 (s, C52), 129.80 (s, C32), 129.59 (s, C54), 129.10 (s, C64), 128.50 (s, C62), 126.38 (d, $^{3}J(^{13}C–^{31}P)$ = 10 Hz, C12), 121.24 (d, $^{1}J(^{13}C–^{31}P)$ = 92 Hz, C21), 118.82 (d, $^{1}J(^{13}C–^{31}P)$ = 92 Hz, C10), 115.71 (d, $^{1}J(^{13}C–^{31}P)$ = 75 Hz, C40), 32.72 (d, $^{1}J(^{13}C–^{31}P)$ = 46 Hz, C1), 23.33 (s, C28), 22.52 (s, C68), 22.10 (s, C38), 21.96 (s, C56), 21.30 (s, C57), 21.17 (s, C37), 21.13 (s, C66, C67), 20.93 (s, C36), 20.80 (d, $^{3}J(^{13}C–^{31}P)$ = 6 Hz, C26), 19.84 (s, C58), 19.64 (d, $^{4}J(^{13}C–^{31}P)$ = 2 Hz, C27). **^{31}P{1H} NMR (243 MHz, CD_2Cl_2)**: δ = 31.95 (s). **HRMS ESI (m/z):** $[M]^+$ calculated. for $C_{49}H_{51}PCl$, 705.34114; found, 705.34092.

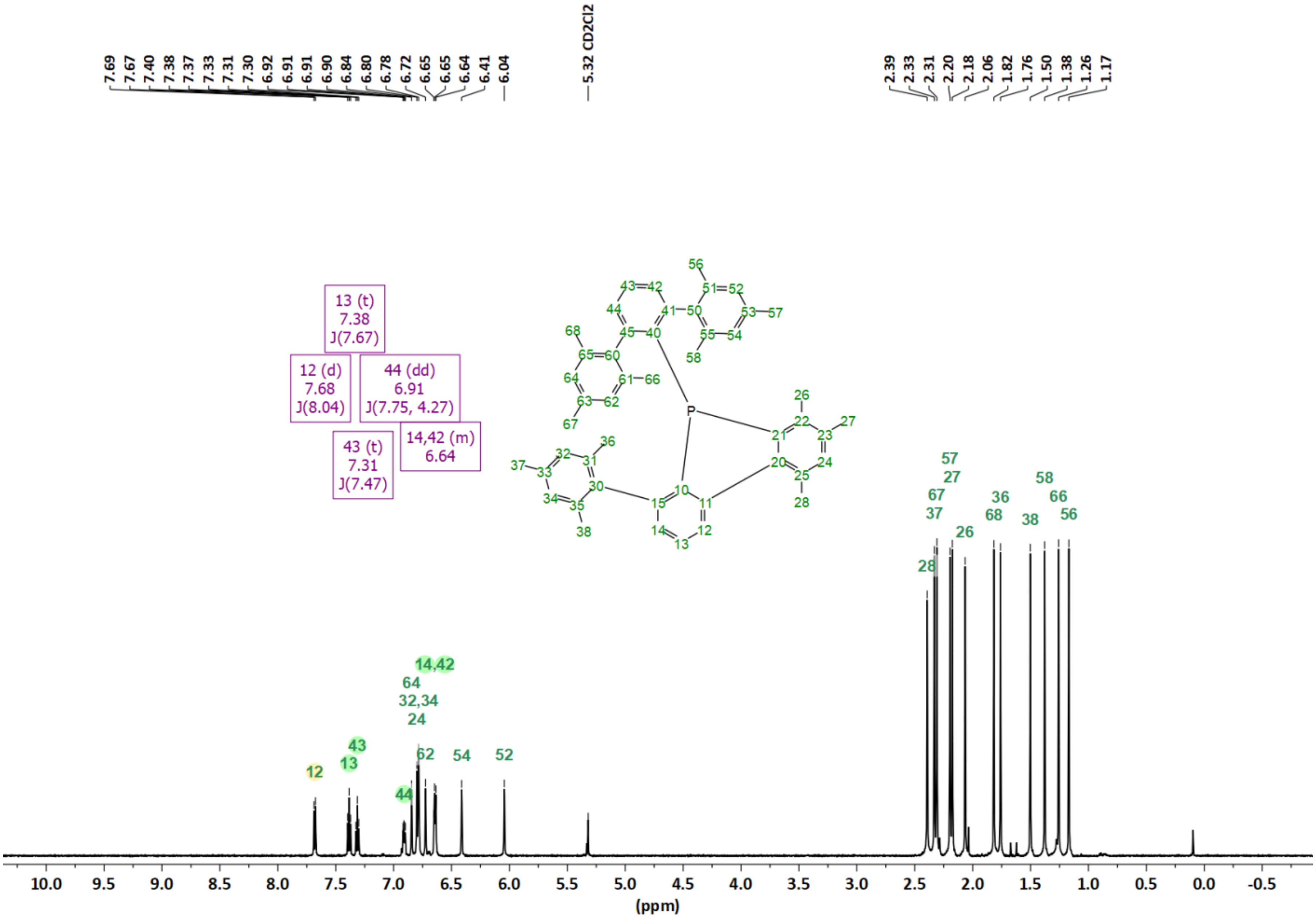

Figure S4. ^{1}H NMR (CD_2Cl_2, 600 MHz) spectrum of **3a**.

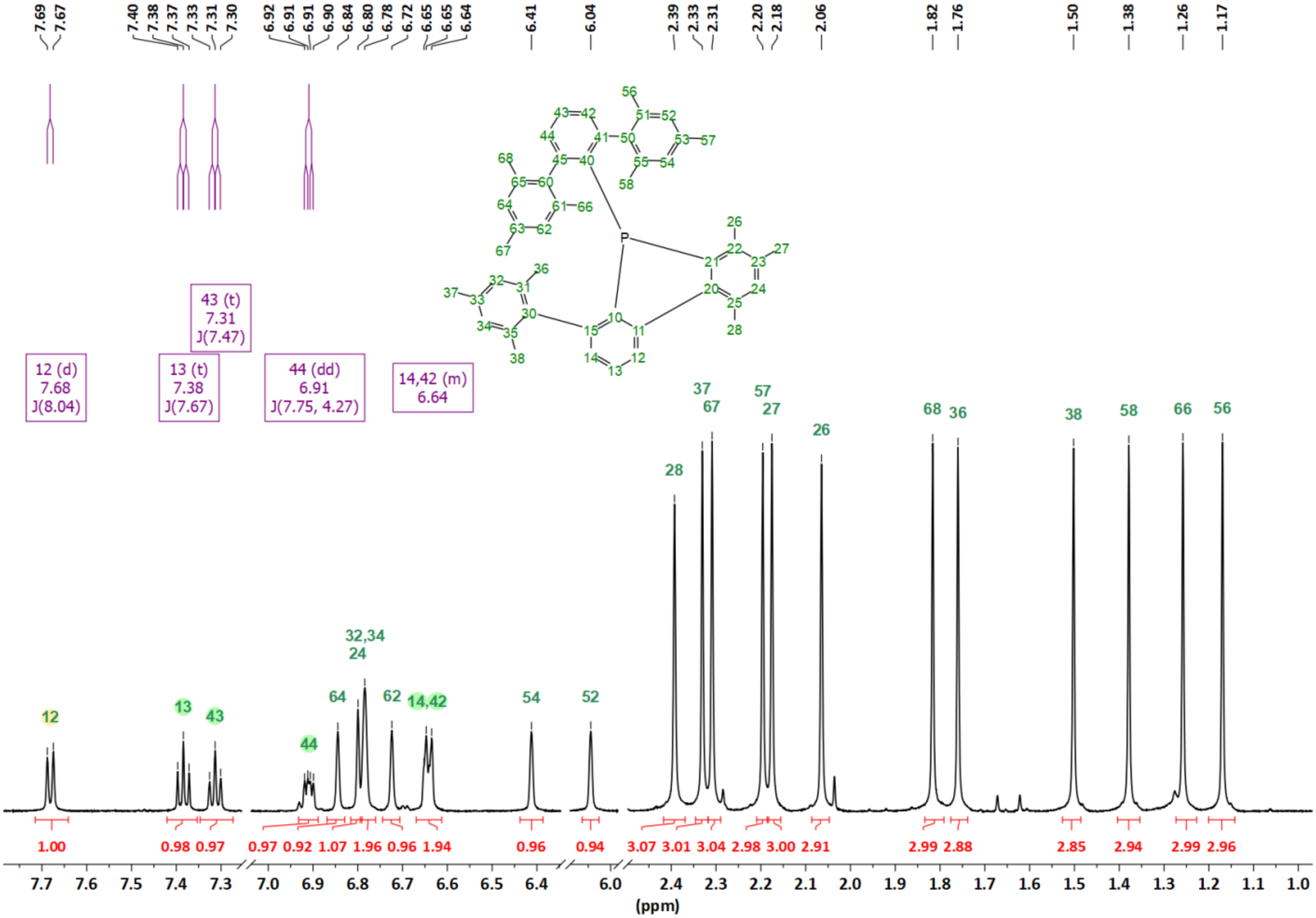

Figure S5. ^{1}H NMR (CD_2Cl_2, 600 MHz) spectrum (detail) of **3a**.

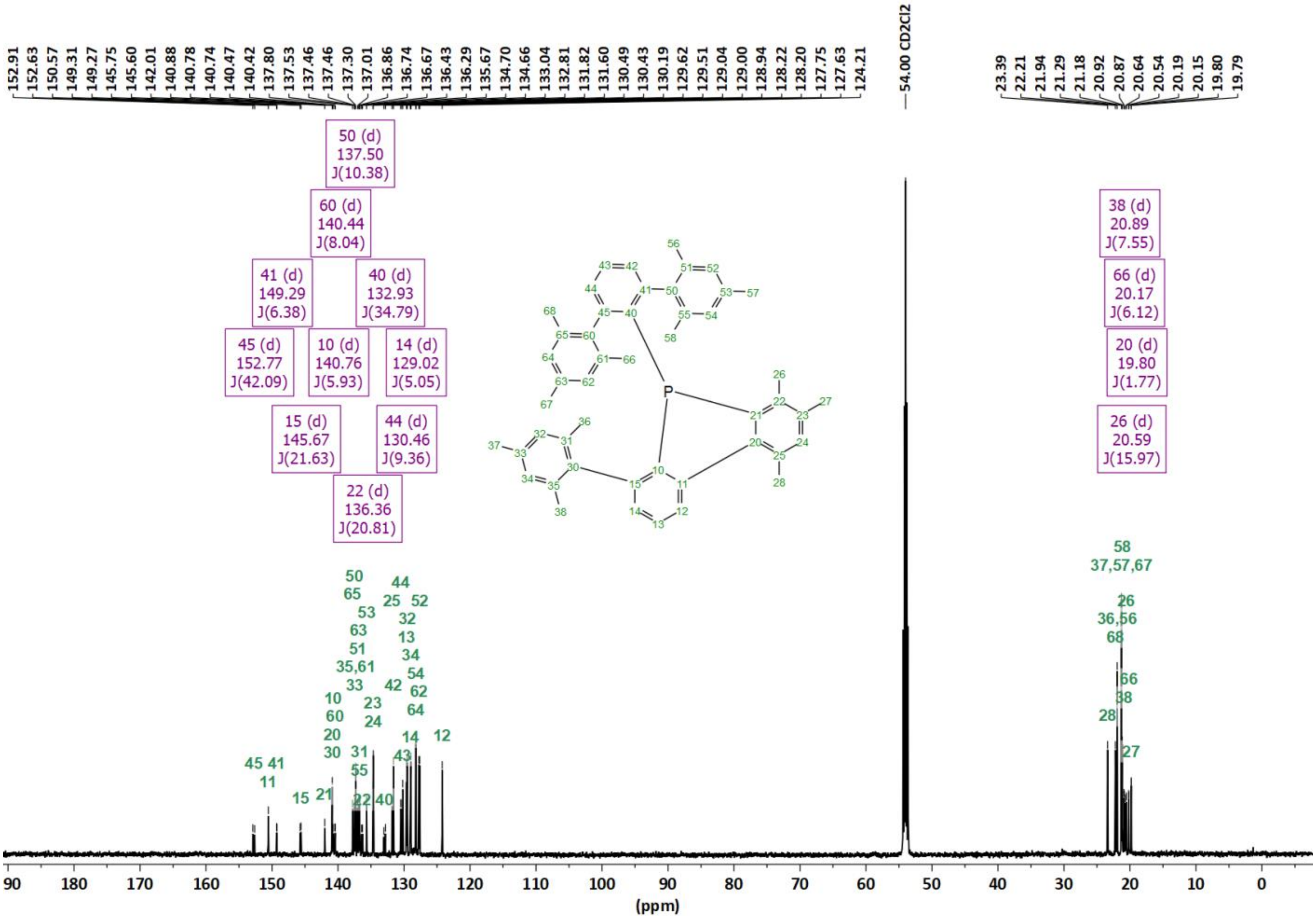

Figure S6. ^{13}C NMR (CD_2Cl_2, 151 MHz) spectrum of **3a**.

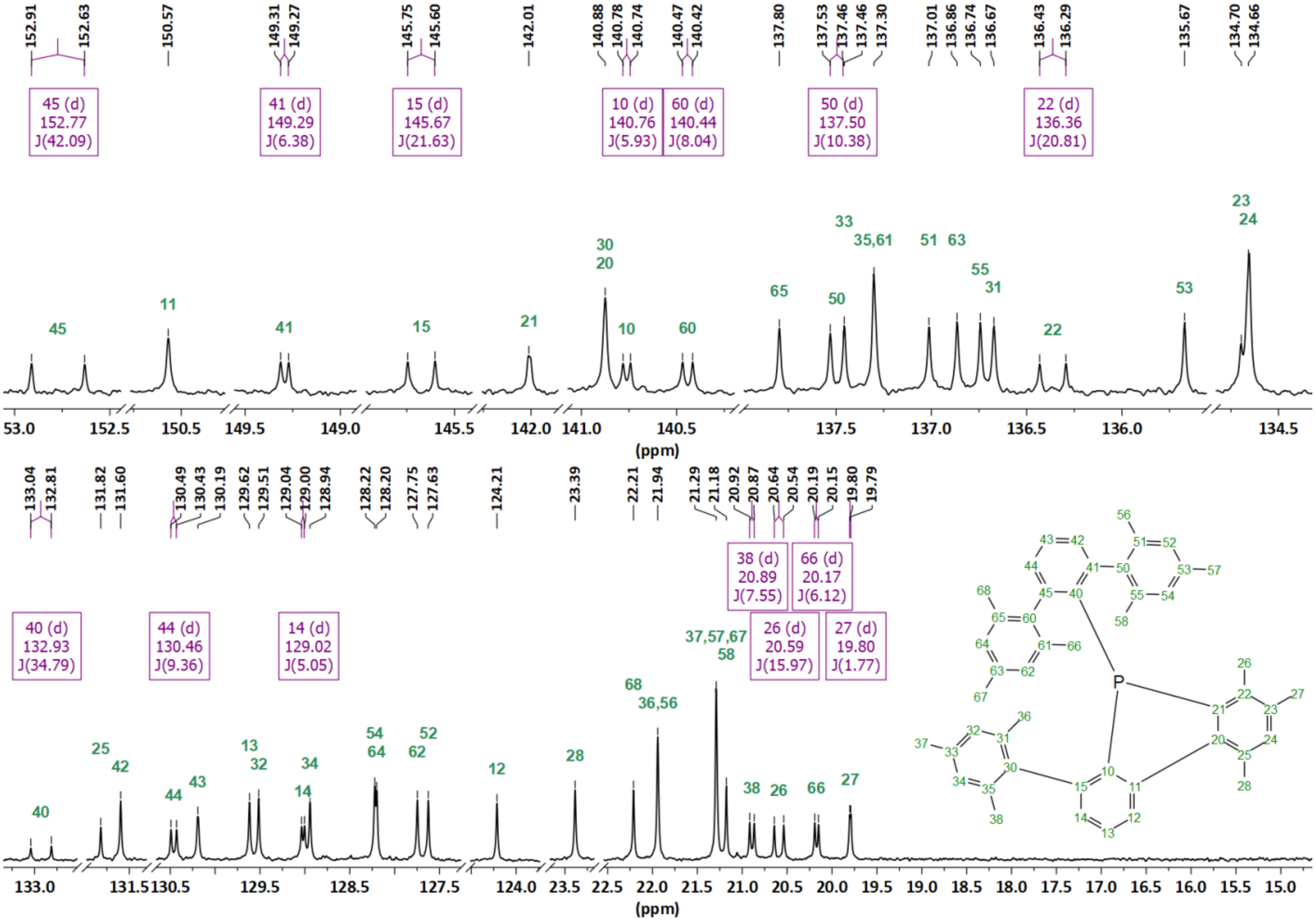

Figure S7. ^{13}C NMR (CD_2Cl_2, 151 MHz) spectrum (detail) of **3a**.

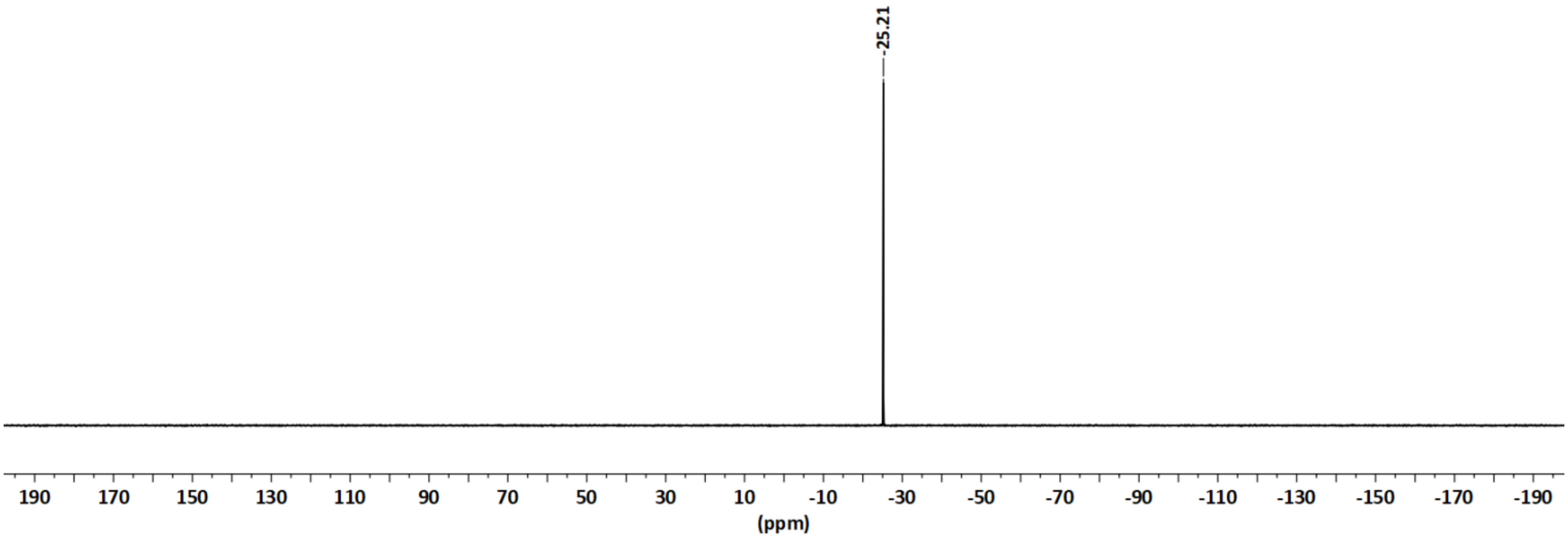

Figure S8. $^{31}P\{1H\}$ NMR (CD_2Cl_2, 243 MHz) spectrum of **3a**.

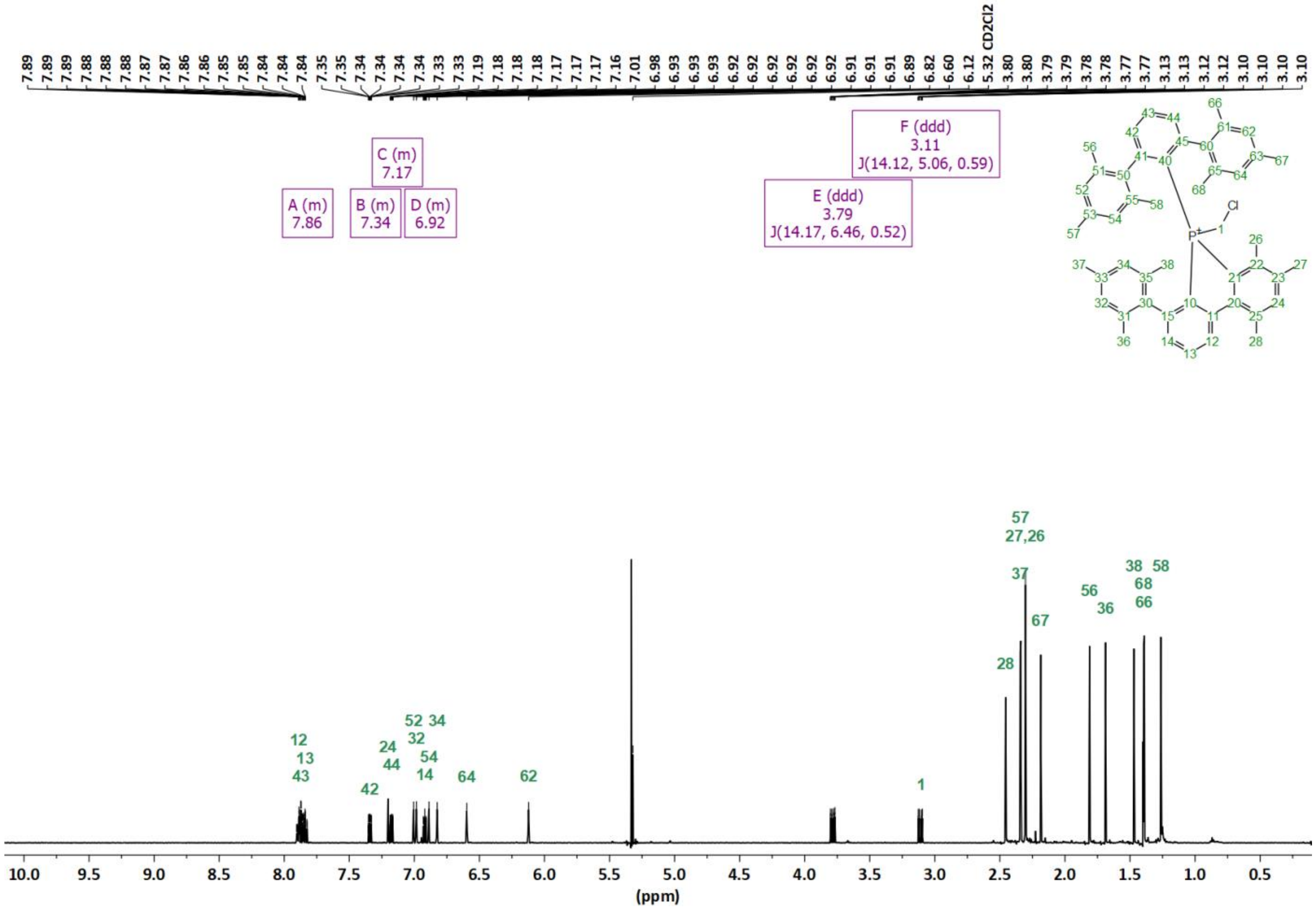

Figure S9. ^1H NMR (CD_2Cl_2, 600 MHz) spectrum of [**4**][$AlCl_4$].

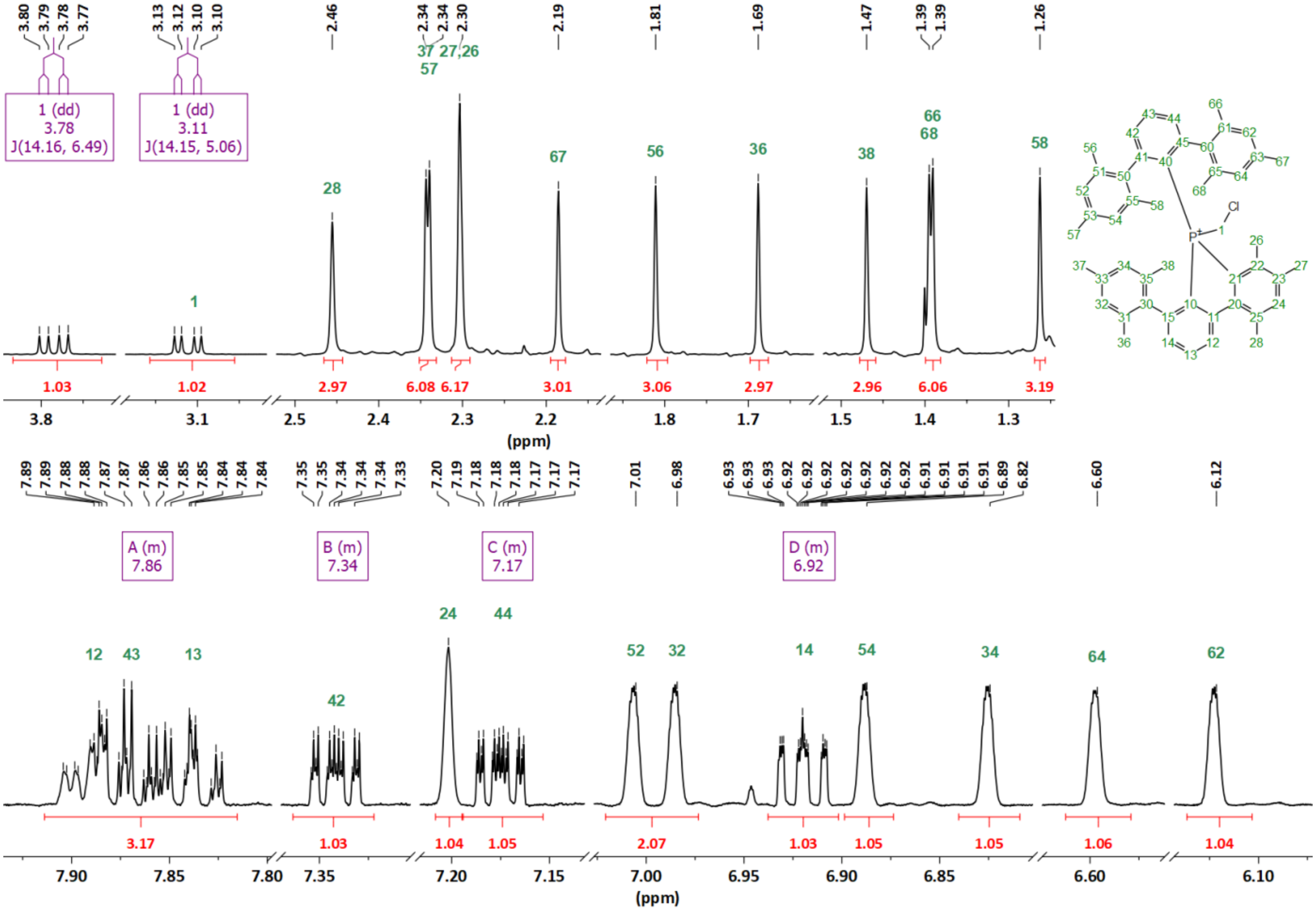

Figure S10. ^{1}H NMR (CD_2Cl_2, 600 MHz) spectrum (detail) of [**4**][$AlCl_4$].

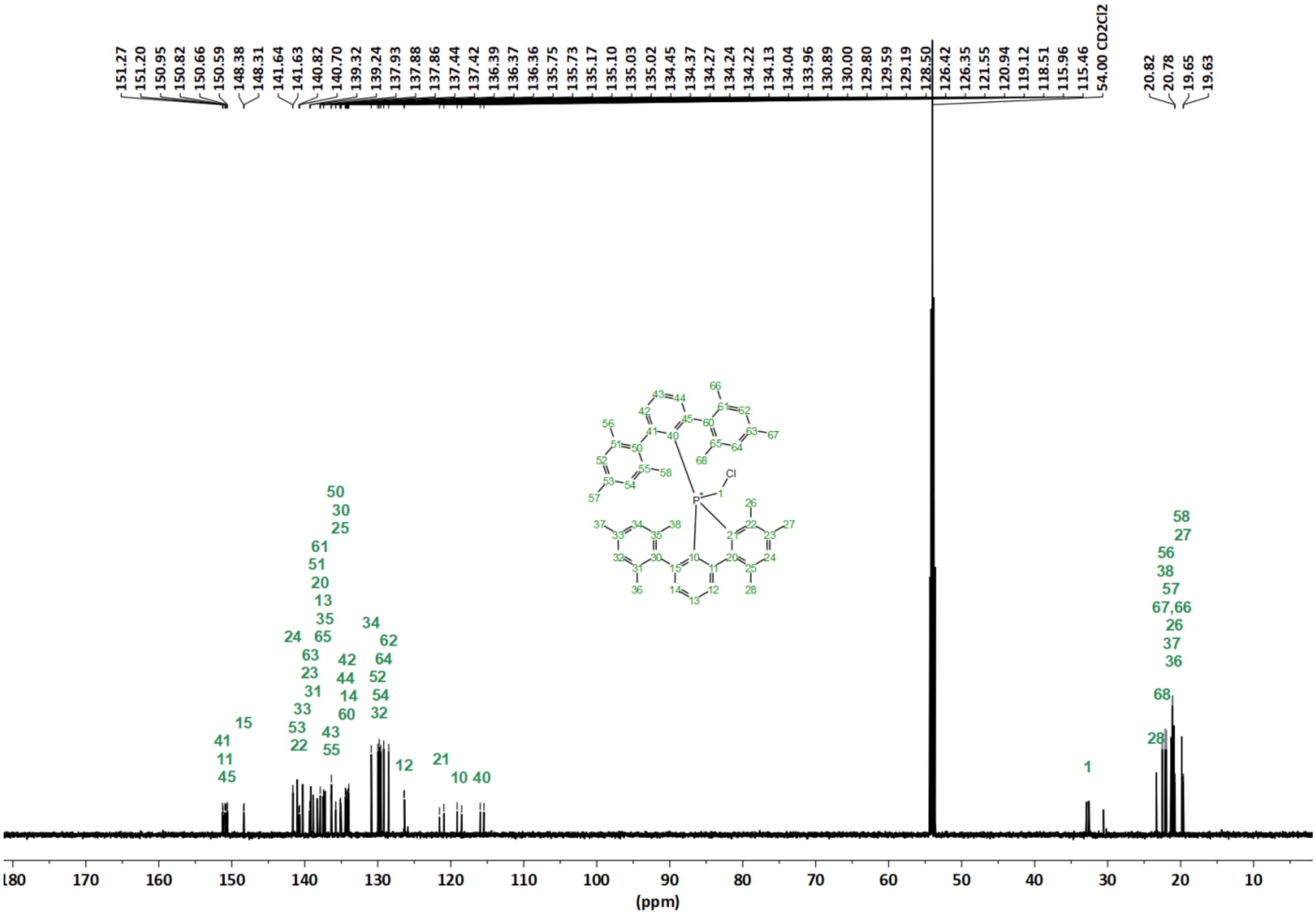

Figure S11. ^{13}C NMR (CD_2Cl_2, 151 MHz) spectrum of [**4**][$AlCl_4$].

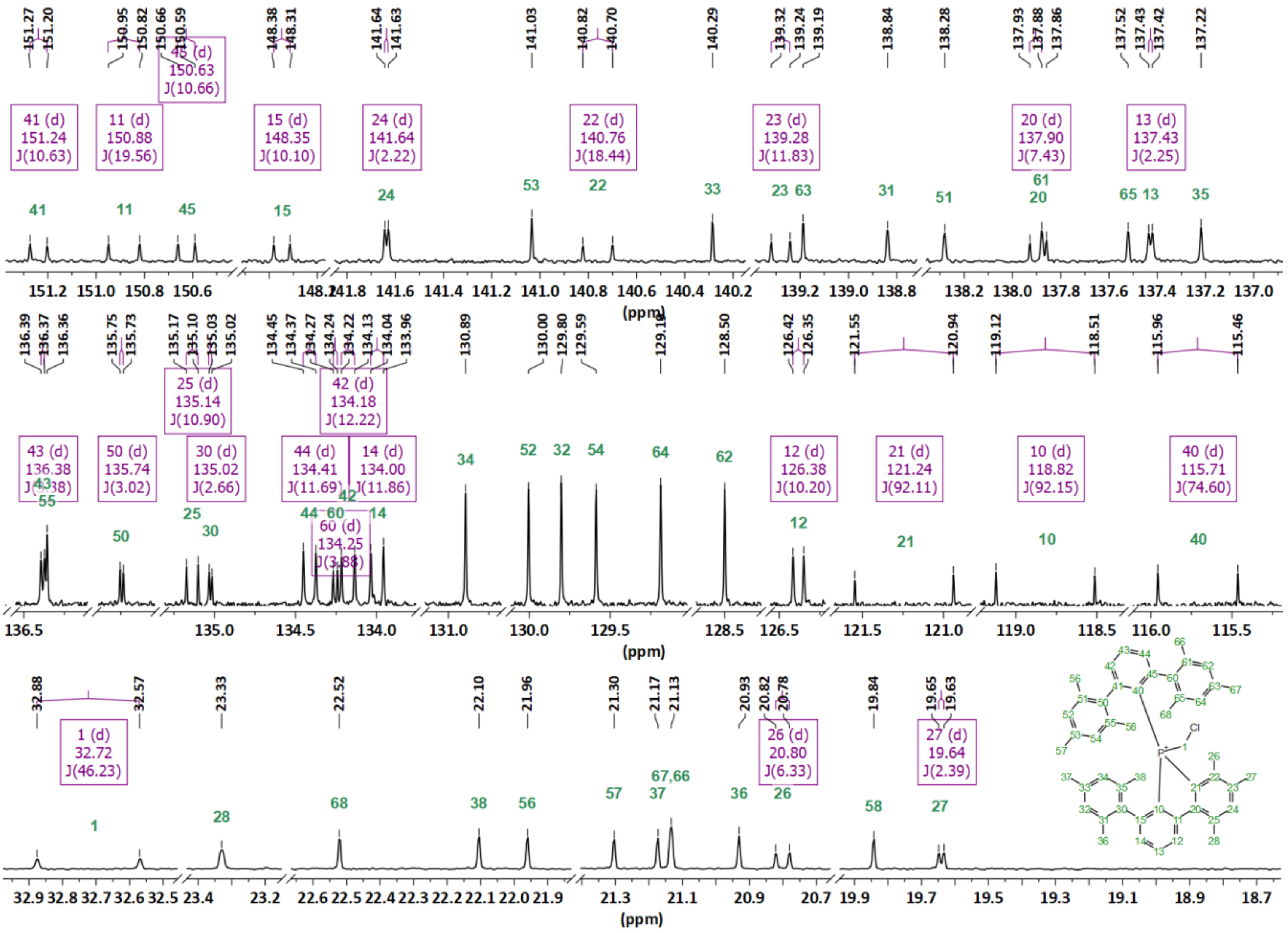

Figure S12. ^{13}C NMR (CD_2Cl_2, 151 MHz) spectrum of [**4**][$AlCl_4$].

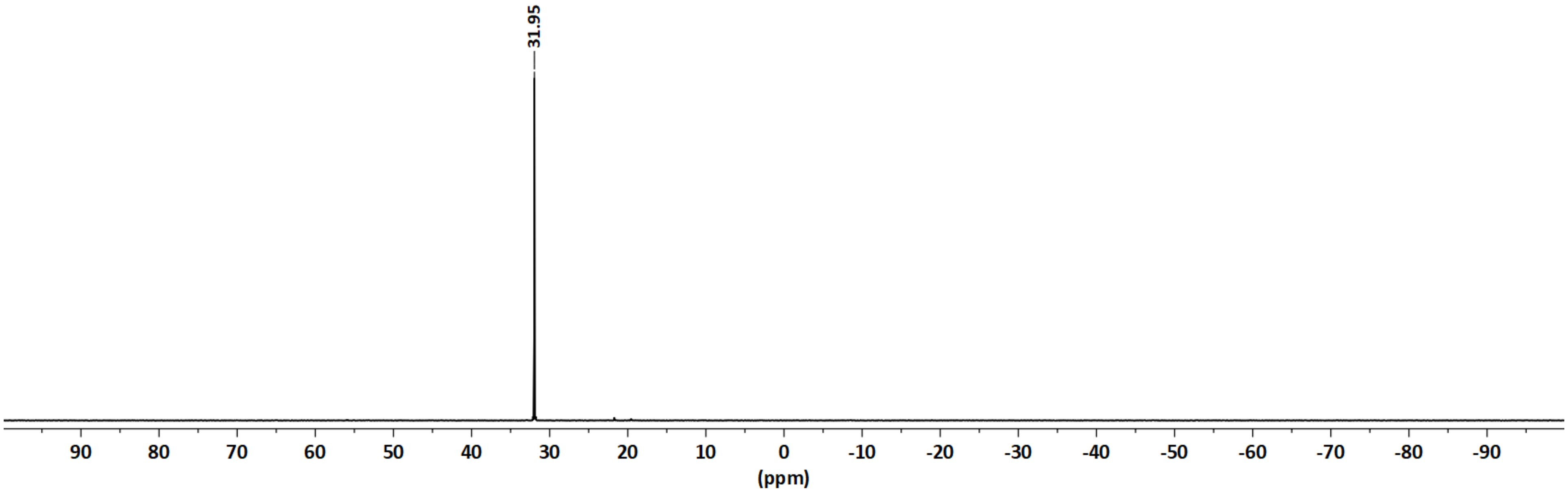

Figure S13. $^{31}P\{^1H\}$ NMR (CD_2Cl_2, 243 MHz) spectrum of [**4**][$AlCl_4$].

Synthesis and characterization of 3b

A 50 mL Schlenk tube fitted with a J. Young valve was charged under argon atmosphere with (2,6-$Mes_2C_6H_3)_2AsF$ (100 mg, 0.14 mmol) and [Et_3Si(toluene)][$B(C_6F_5)_4$] (150 mg, 0.16 mmol). 1,2-difluorobenzene (4 mL) was added over the solid mixture at room temperature. The initially dark red solution turned dark yellow after a few seconds. After 1 h, triethylamine (0.1 mL, 0.72 mmol) was added and the resulting light yellow solution was stirred for 1 h. All volatiles were removed under reduced pressure. Hexane (10 mL), was added and the suspension was filtered through a PTFE syringe filter. The solvent was allowed to slowly evaporate (in a glovebox) to give **3b** as a colourless crystalline solid (50 mg, 54 %).

^{1}H NMR (600 MHz, CD_2Cl_2): δ = 7.74 (d, $^3J(^1H–^1H)$ = 8 Hz, 1H, H12), 7.41 (d, $^3J(^1H–^1H)$ = 7 Hz, 1H, H13), 7.32 (t, $^3J(^1H–^1H)$ = 8 Hz, 1H, H13), 7.32 (t, $^3J(^1H–^1H)$ = 8 Hz, 1H, H43), 6.94 (dd, $^3J(^1H–^1H)$ = 8 Hz, $^4J(^1H–^1H)$ = 1 Hz, 1H, H44), 6.92 (s, 1H, H62), 6.86 (s, 1H, H34), 6.84 (s, 1H, H32), 6.84 (s, 1H, H24), 673 (d, $^3J(^1H–^1H)$ = 7 Hz, 1H, H14), 6.73 (s, 1H, H64), 6.65 (dd, $^3J(^1H–^1H)$ = 8 Hz, $^4J(^1H–^1H)$ = 1 Hz, 1H, H42), 6.44 (s, 1H, H54), 6.10 (s, 1H, H52), 2.46 (s, 3H, H28), 2.46 (s, 3H, H37), 2.37 (s, 3H, H67), 2.25 (s, 3H, H57), 2.23 (s, 3H, H27), 2.22 (s, 3H, H26), 1.97 (s, 3H, H66), 1.95 (s, 3H, H38), 1.62 (s, 3H, H36), 1.43 (s, 3H, H58), 1.29 (s, 3H, H68), 1.27 (s, 3H, H56). **$^{13}C\{^1H\}$ NMR (151 MHz, CD_2Cl_2)**: δ = 152.24 (s, C15), 151.07 (s, C41), 148.87 (s, C45), 146.29 (s, C21), 145.91 (s, C11), 144.24 (s, C10), 142.64 (s, C20), 141.03 (s, C30), 140.80 (s, C60), 137.68 (s, C50), 137.44 (s, C33), 137.25 (s, C31), 137.09 (s, C61), 137.07 (s, C40), 136.80 (s, C51, C63), 136.68 (s, C23), 136.59 (s, C55), 136.56 (s, C35), 135.48 (s, C53), 135.12 (s, C24), 134.49 (s, C22), 132.60 (s, C25), 131.49 (s, C42), 129.88 (s, C44), 129.70 (s, C34), 129.62 (s, C43), 129.47 (s, C13), 128.97 (s, C32), 128.69 (s, C14) 128.20 (s, C62), 128.19 (s, C54), 125.16 (s, C12), 24.12 (s, C28), 22.48 (s, C26), 22.08 (s, C38), 21.97 (s, C66), 21.89 (s, C56), 21.48 (s, 57), 21.41 (s, C37, C67), 21.26 (s, C36), 21.20 (s, C58), 20.56 (s, C68), 20.00 (s, C27). **HRMS ESI (m/z):** $[M+Na]^+$ calculated. for $C_{48}H_{49}AsNa$, 723.29424; found, 723.29385.

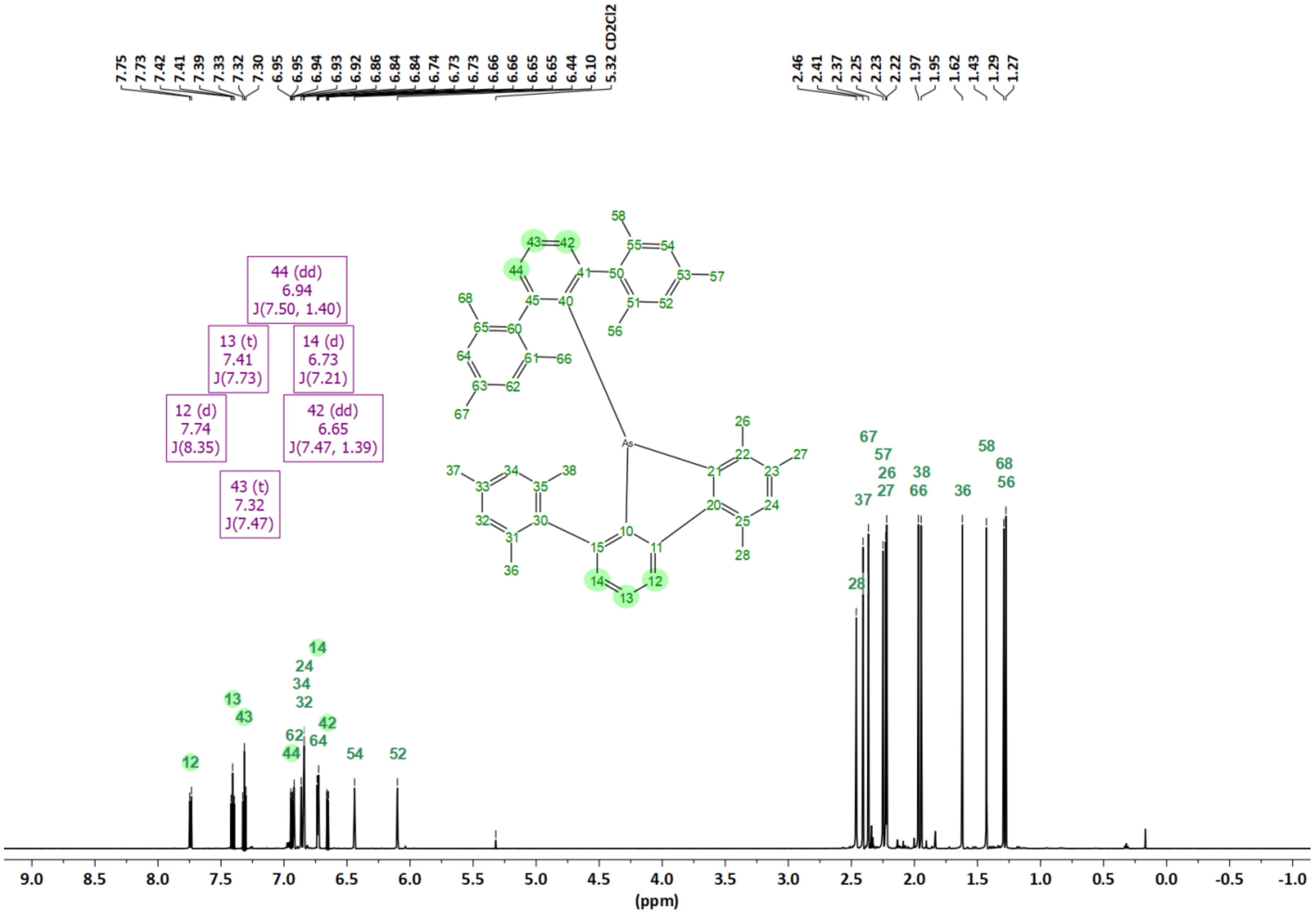

Figure S14. ^{1}H NMR (CD_2Cl_2, 600 MHz) spectrum of **3b**.

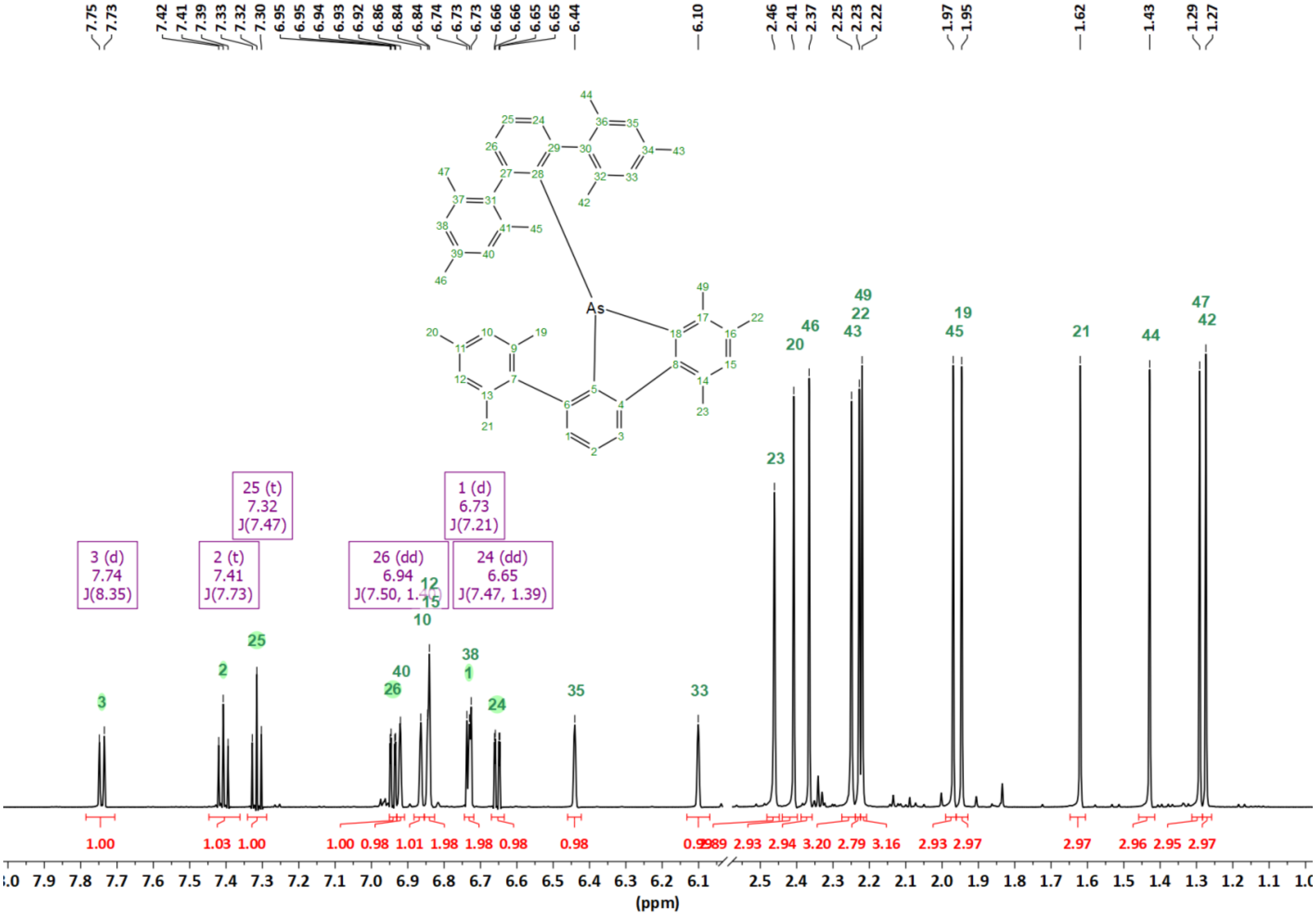

Figure S15. ^{1}H NMR (CD_2Cl_2, 600 MHz) spectrum (detail) of **3b**.

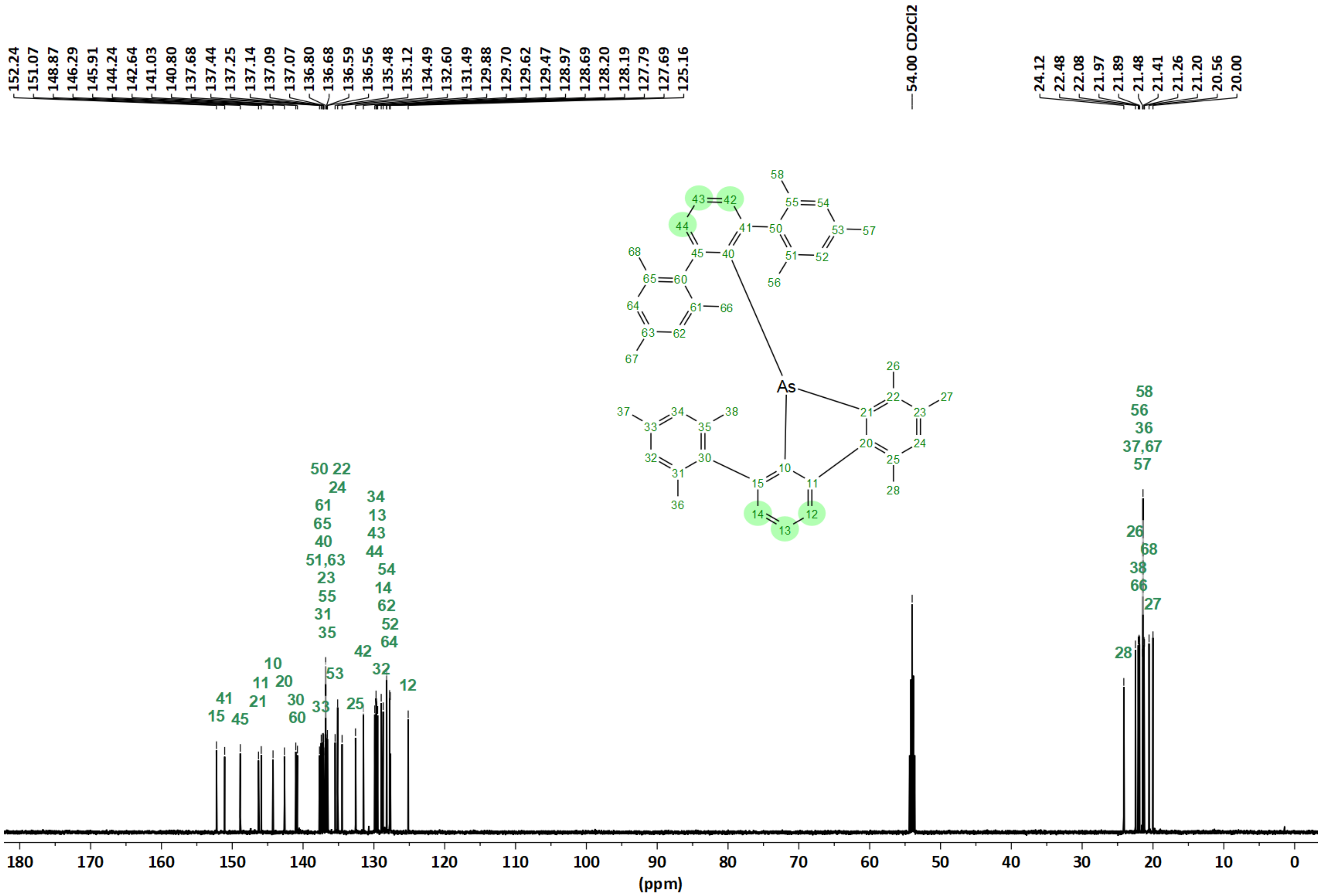

Figure S16. ^{13}C NMR (CD_2Cl_2, 151 MHz) spectrum of **3b**.

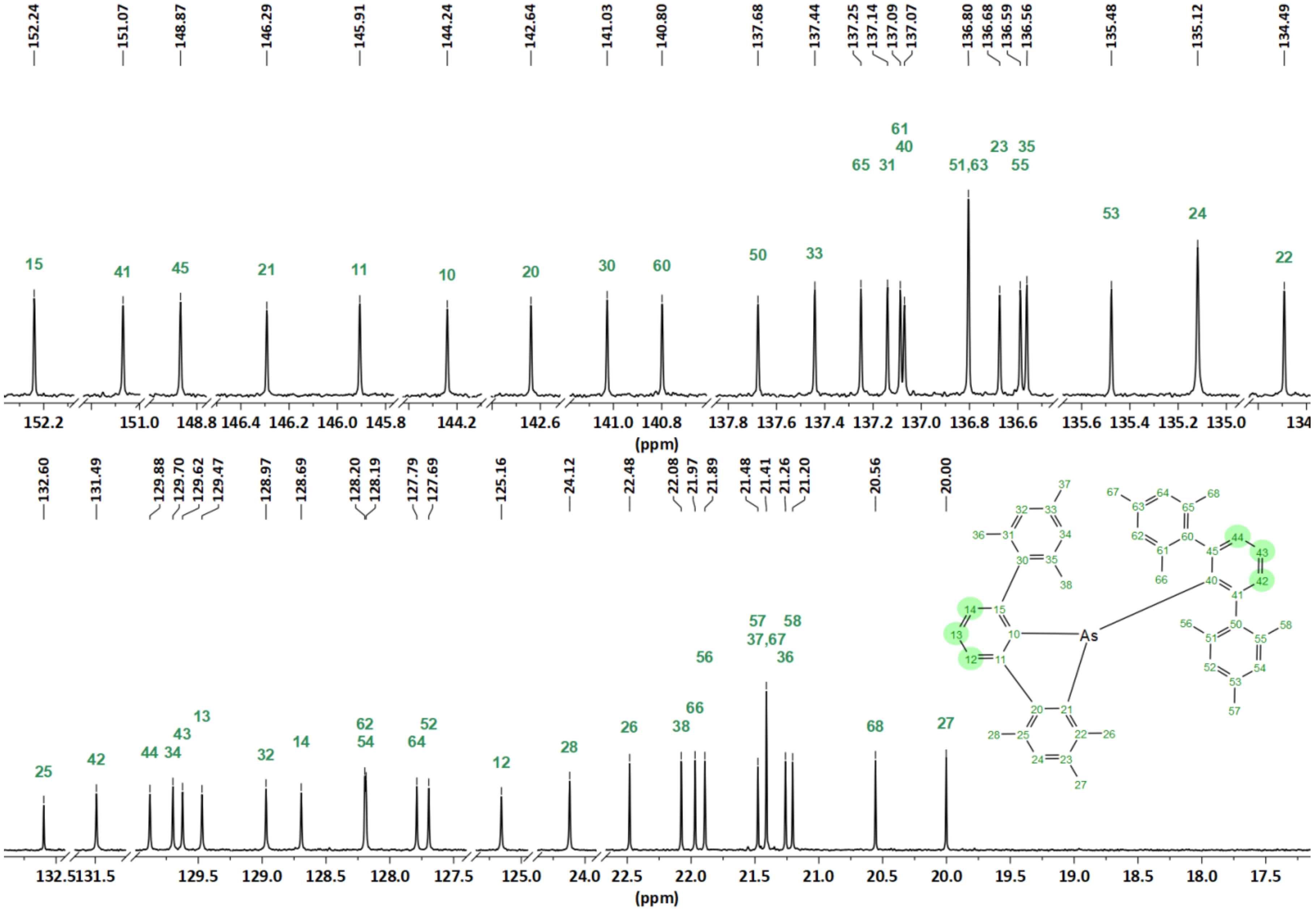

Figure S17. ^{13}C NMR (CD_2Cl_2, 151 MHz) spectrum (detail) of **3b**.

Synthesis and characterization of [2a][BAr$^F_4$]

A 50 mL Schlenk tube fitted with a J. Young valve was charged under argon atmosphere with **3a** (50 mg, 0.076 mmol) and NaBAr$^F_4$ (67 mg, 0.076 mmol) and CH_2Cl_2 (3 mL). To the resulting solution, HCl in Et_2O (1.7 M, 0.1 mL, 0.17 mmol) was added. After 5 minutes of stirring, all volatiles were evaporated at reduced pressure. CH_2Cl_2 was added and the turbid solution was filtered through a PTFE syringe filter. The solvent was evaporated under reduced pressure. The remaining solid was washed with 2×4 mL hexane and dried at 80 °C ($2 \cdot 10^{-2}$ mbar). Compound [**2a**][BAr$^F_4$] (110 mg, 96%) was obtained as a white powder.

^{1}H NMR (600 MHz, CD_2Cl_2): δ = 7.82 (td, $^3J(^1H–^1H)$ = 8 Hz, $^5J(^1H–^{31}P)$ = 2 Hz, 1H, H43), 7.75 (m, 10 H, *o*-H-Ar^F, H12, H13), 7.57 (s, 4H, *p*-H-Ar^F), 7.42 (d, $^1J(^1H–^{31}P)$ = 500 Hz, 1H, PH), 7.33 (ddd, $^3J(^1H–^1H)$ = 7 Hz, $^4J(^1H–^{31}P)$ = 6 Hz, $^4J(^1H–^1H)$ = 1 Hz, 1H, H43), 7.22 (s, 1H, H24), 7.17 (m, 1H, H42), 7.01 (s, 1H, H62), 7.00 (m, 2H, H32, H14), 6.82 (s, 1H, H34), 6.66 (s, 1H, H64, 6.54 (s, 1H, H54), 6.31 (s, 1H, H52), 2.42 (s, 6H, H28, H37), 2.32 (s, 3H, H67), 2.26 (s, 3H, H57), 2.23 (s, 3H, H27), 2.16 (s, 3H, H26), 2.04 (s, 3H, H66), 2.03 (s, 3H, H36), 1.56 (s, 3H, 58), 1.49 (s, 3H, H38), 1.33 (s, 3H, H56). **^{13}C{^{1}H} NMR (151 MHz, CD_2Cl_2)**: δ = 162.4 (m, *i*-C-Ar^F), 151.67 (d, $^2J(^{13}C–^{31}P)$ = 13 Hz, C45), 150.02 (d, $^2J(^{13}C–^{31}P)$ = 11 Hz, C41), 149.93 (d, $^2J(^{13}C–^{31}P)$ = 21 Hz, C11), 147.84 (d, $^2J(^{13}C–^{31}P)$ = 10 Hz, C15), 142.16 (d, $^4J(^{13}C–^{31}P)$ = 2 Hz, C24), 141.05 (d, $^2J(^{13}C–^{31}P)$ = 20 Hz, C20), 140.40 (s, C33), 139.94 (s, C63), 139.51 (d, $^3J(^{13}C–^{31}P)$ = 10 Hz, C23), 139.51 (d, $^2J(^{13}C–^{31}P)$ = 12 Hz, C22), 139.19 (s, C53), 137.42 (s, C51), 137.20 (s, C55), 136.99 (s, C65), 136.92 (d, $^4J(^{13}C–^{31}P)$ = 2 Hz, C13), 136.79 (s, C61), 136.63 (s, C31), 136.51 (d, $^4J(^{13}C–^{31}P)$ = 3 Hz), 136.23 (d, $^3J(^{13}C–^{31}P)$ = 3 Hz C30), 136.22 (s, C35), 135.55 (d, $^3J(^{13}C–^{31}P)$ = 11 Hz, C25), 135.40 (s, br, *o*-C-Ar^F), 134.05 (d, $^3J(^{13}C–^{31}P)$ = 7 Hz, C60), 133.77 (d, $^3J(^{13}C–^{31}P)$ = 12 Hz, C42), 133.73 (d, $^3J(^{13}C–^{31}P)$ = 5 Hz, C50), 133.57 (d, $^3J(^{13}C–^{31}P)$ = 12 Hz, C14), 133.17 (d, $^3J(^{13}C–^{31}P)$ = 10 Hz, C44), 130.96 (s, C32), 130.25 (s, C34), 129.76 (s, C62), 129.49 (qq, $^2J(^{13}C–^{19}F)$ = 31 Hz, $^4J(^{13}C–^{19}F)$ = 3 Hz, *m*-C-Ar^F), 129.40 (s, C64), 129.38 (s, C54), 129.19 (s, C52), 126.13 (d, $^3J(^{13}C–^{31}P)$ = 11 Hz, C12), 125.19 (q, $^1J(^{13}C–^{19}F)$ = 273 Hz, CF_3-Ar^F), 119.21 (d, $^1J(^{13}C–^{31}P)$ = 89.38 Hz, C21), 118.80 (d, $^1J(^{13}C–^{31}P)$ = 91 Hz, C10), 118.06 (septet, $^3J(^{13}C–^{19}F)$ = 4 Hz, *p*-C-Ar^F), 114.35 (d, $^1J(^{13}C–^{31}P)$ = 91 Hz, C40), 22.82 (s, br, C28), 22.16 (s, C66), 22.10 (s, C36), 21.59 (s, C58), 21.57(d, $^3J(^{13}C–^{31}P)$ = 7 Hz, C26), 21.48 (s, C56), 21.46 (s, C37), 21.40 (s, C57), 21.30 (s, C67), 20.56 (s, C68), 20.54 (d, $^5J(^{13}C–^{31}P)$ = 2 Hz, C38), 19.56 (d, $^4J(^{13}C–^{31}P)$ = 2 Hz, C27). **^{31}P NMR (243 MHz, CD_2Cl_2)**: δ = −14.95 (d, $^1J(^{31}P-^1H)$ = 501 Hz). **^{19}F NMR (339 MHz, CD_2Cl_2)**: δ = −62.81 (s). **HRMS ESI (m/z):** $[M+H]^+$ calculated. for $C_{48}H_{50}P$, 657.36418; found, 657.36446.

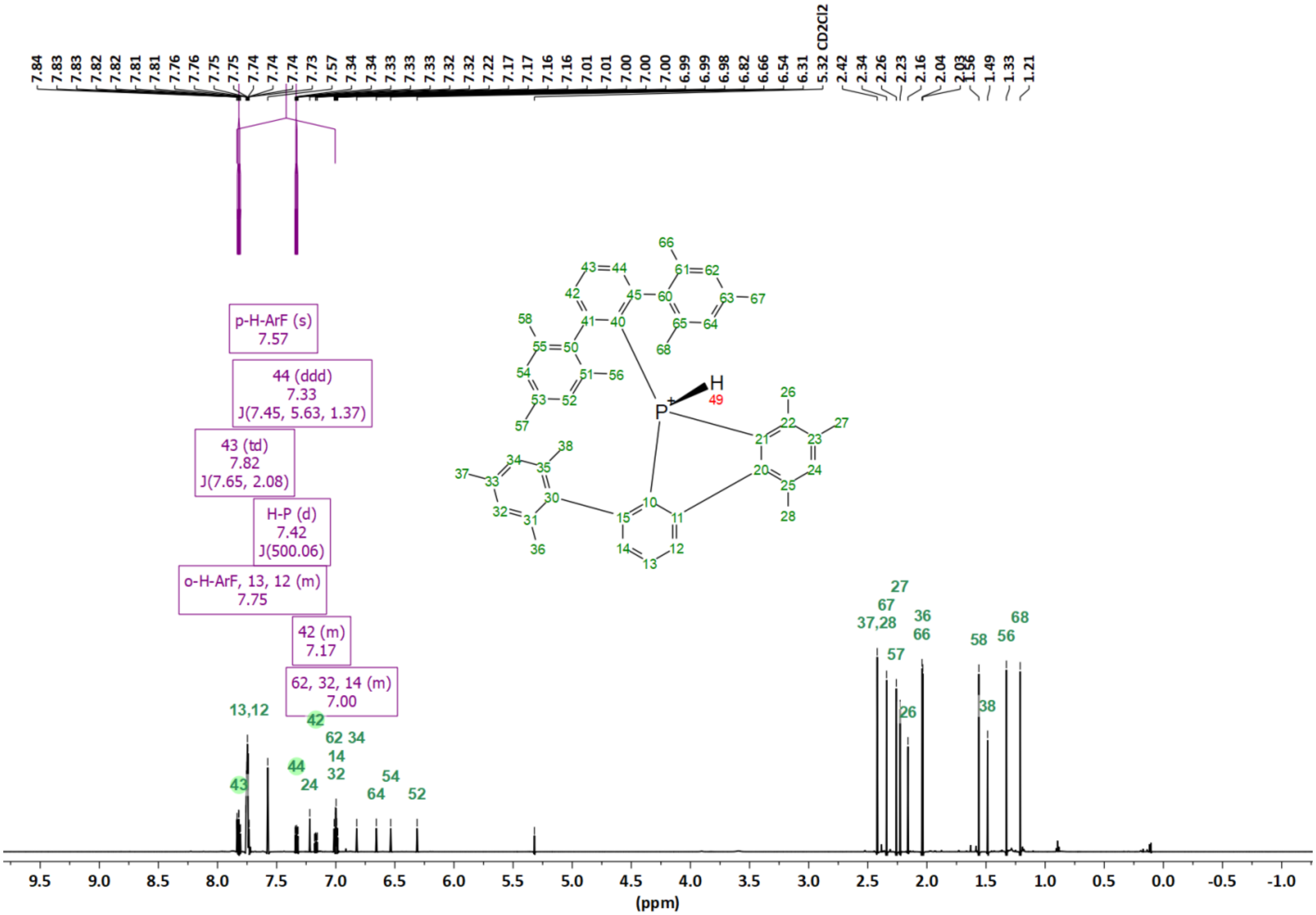

Figure S18. ${}^{1}H$ NMR (CD_2Cl_2, 600 MHz) spectrum of [**2a**][$BAr^F{}_4$].

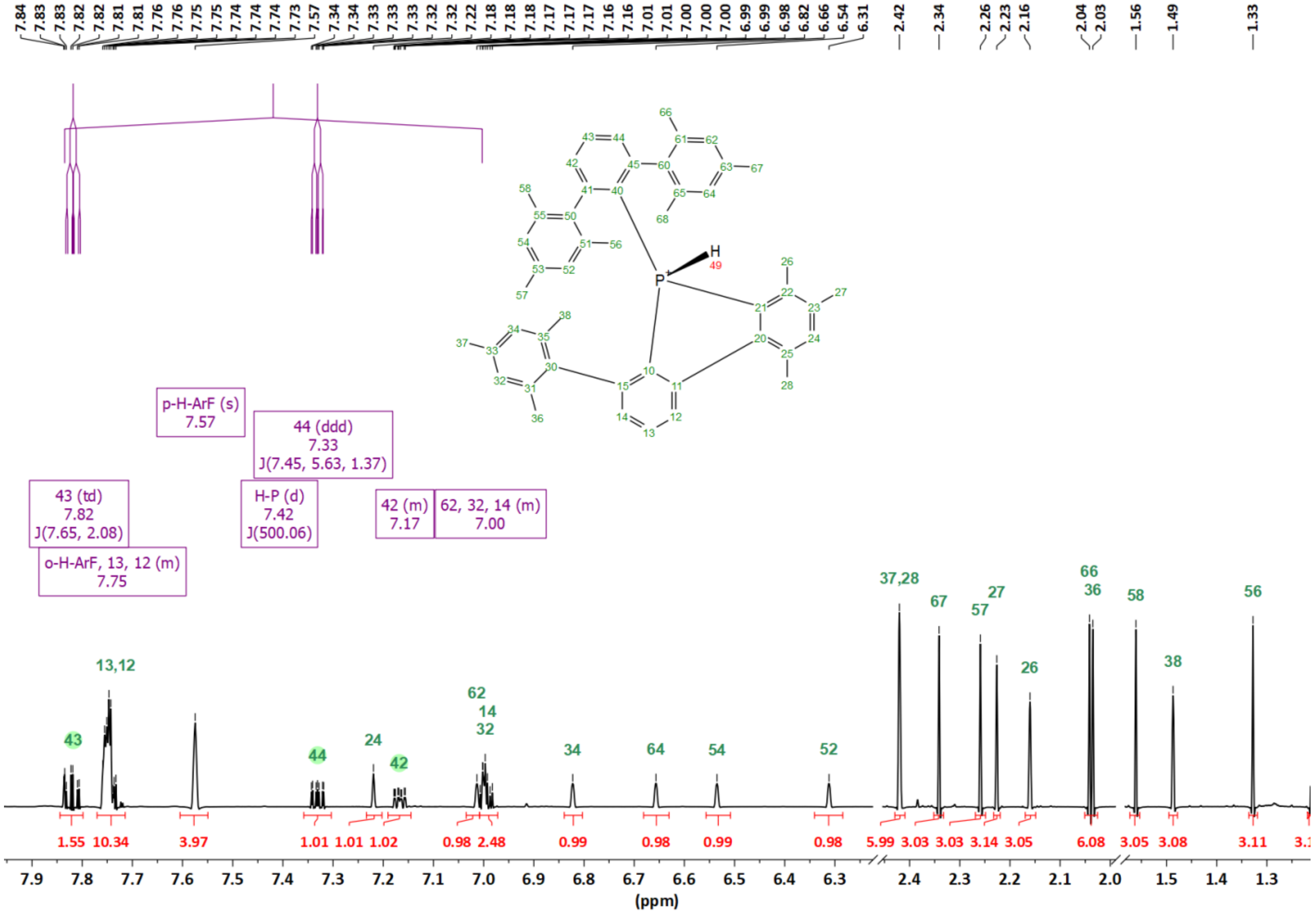

Figure S19. ^{1}H NMR (CD_2Cl_2, 600 MHz) spectrum (detail) of [**2a**][$BAr^F{}_4$].

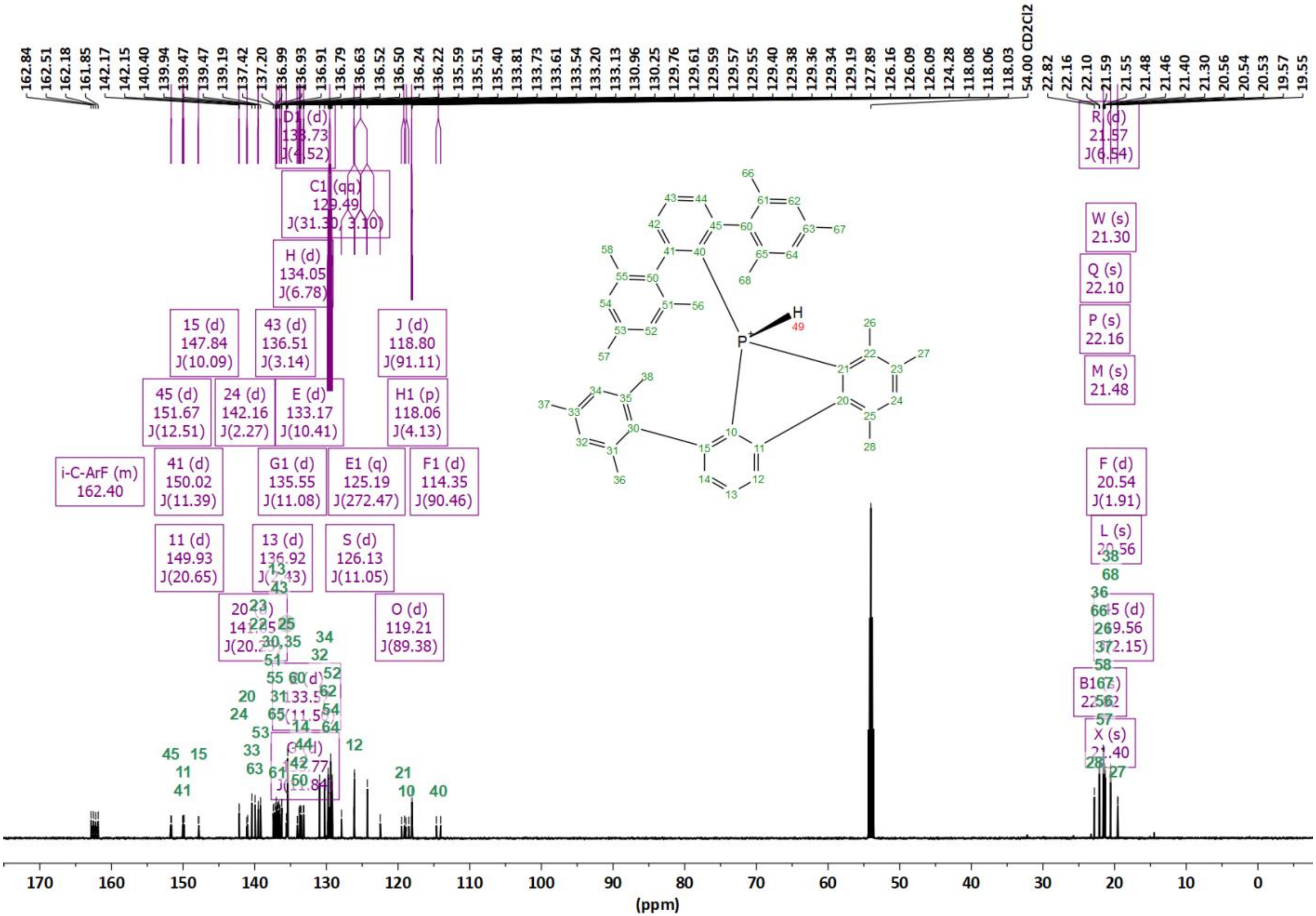

Figure S20. ^{13}C NMR (CD_2Cl_2, 151 MHz) spectrum of [**2a**][BAr^F_4].

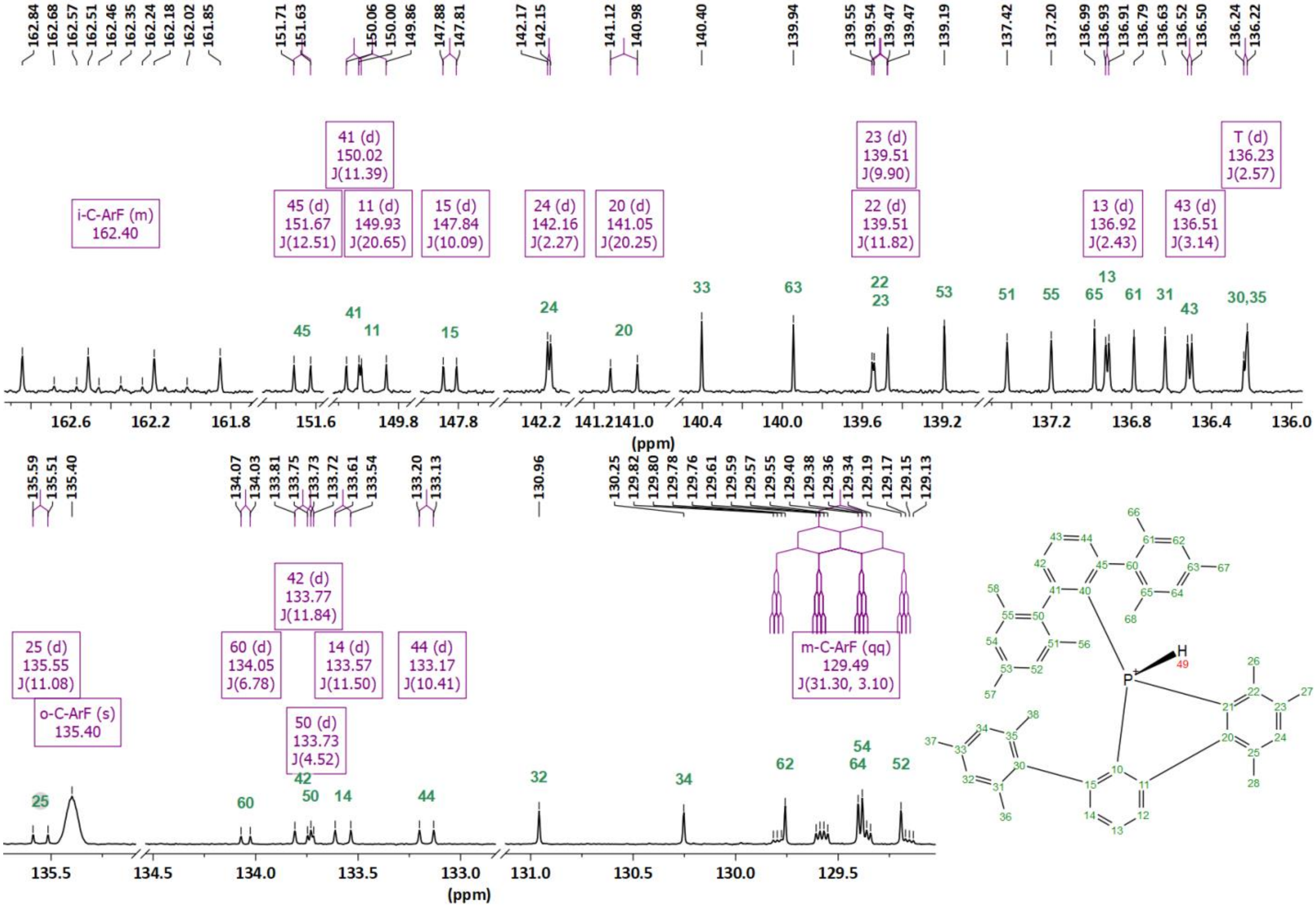

Figure S21. ^{13}C NMR (CD_2Cl_2, 151 MHz) spectrum (detail) of [**2a**][BAr$^F{}_4$].

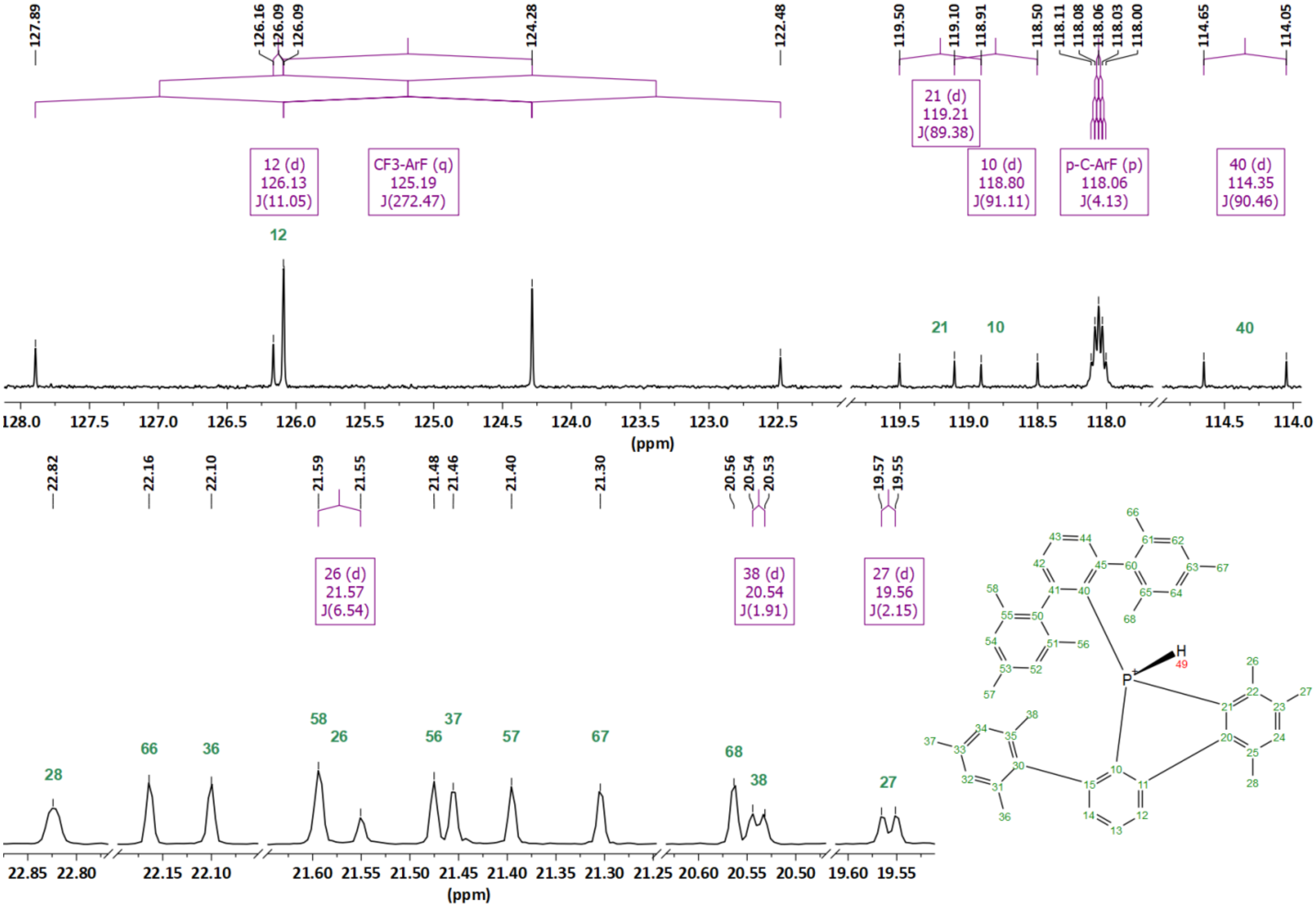

Figure S22. ^{13}C NMR (CD_2Cl_2, 151 MHz) spectrum (detail) of [**2a**][BAr$^F{}_4$].

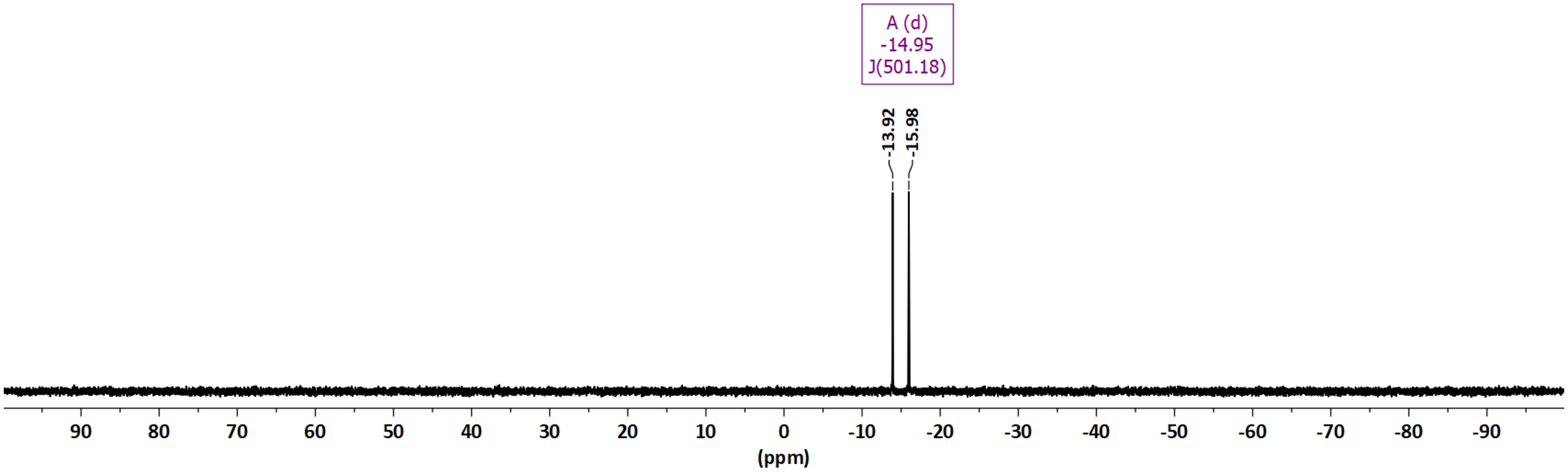

Figure S23. $^{31}P\{^1H\}$ NMR (CD_2Cl_2, 243 MHz) spectrum of [**2a**][BAr$^F{}_4$].

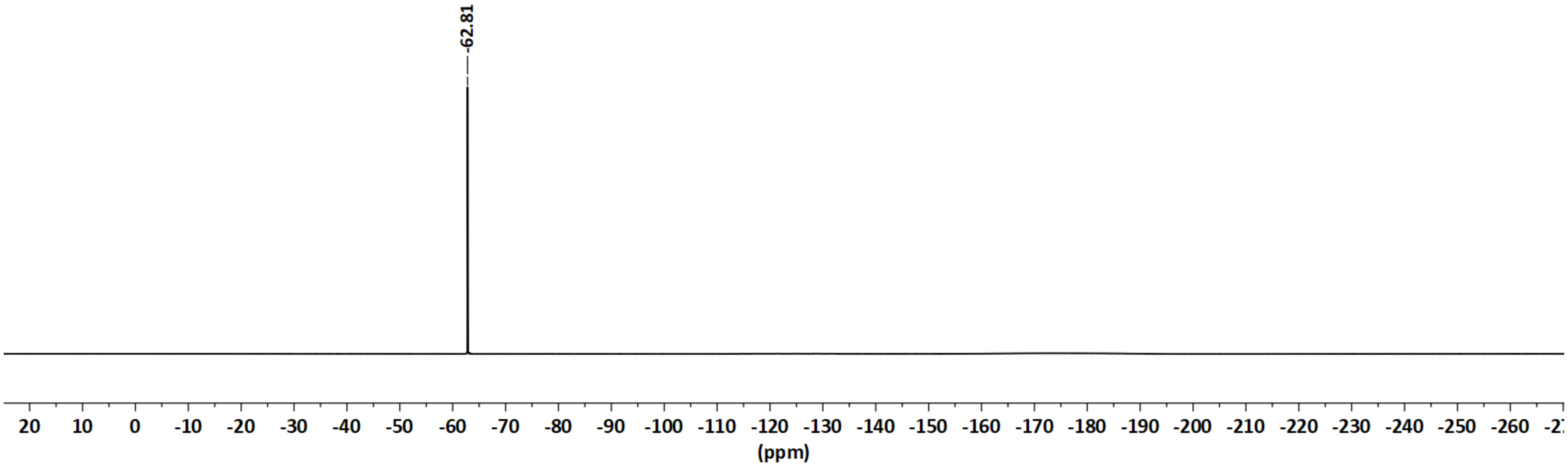

Figure S24. $^{19}F\{^1H\}$ NMR (CD_2Cl_2, 565 MHz) spectrum of [**2a**][BAr$^F{}_4$].

X-Ray diffraction studies

Intensity data of (2,6-$Mes_2C_6H_3)_2AsF$, [**2a**][$BAr^F{}_4$], **3a**, **3b** and [**4**][$AlCl_4$] was collected on a Bruker Venture D8 diffractometer at 100 K with graphite-monochromated Mo-Kα (0.7107 Å) radiation. All structures were solved by direct methods and refined based on F^2 by use of the SHELX program package[S7] as implemented in WinGX and OLEX.[S8] All non-hydrogen atoms were refined using anisotropic displacement parameters. Hydrogen atoms attached to carbon atoms were included in geometrically calculated positions using a riding model. Diffuse electron density due to heavily disordered solvent molecules was account for **3a** using the SQUEEZE routine.[S9] Crystal and refinement data are collected in Tables S1 and S2. Figures were created using DIAMOND.[S10] Crystallographic data for the structural analyses have been deposited with the Cambridge Crystallographic Data Centre. Copies of this information may be obtained free of charge from The Director, CCDC, 12 Union Road, Cambridge CB2 1EZ, UK (Fax: +44-1223-336033; e-mail: deposit@ccdc.cam.ac.uk or http://www.ccdc.cam.ac.uk).

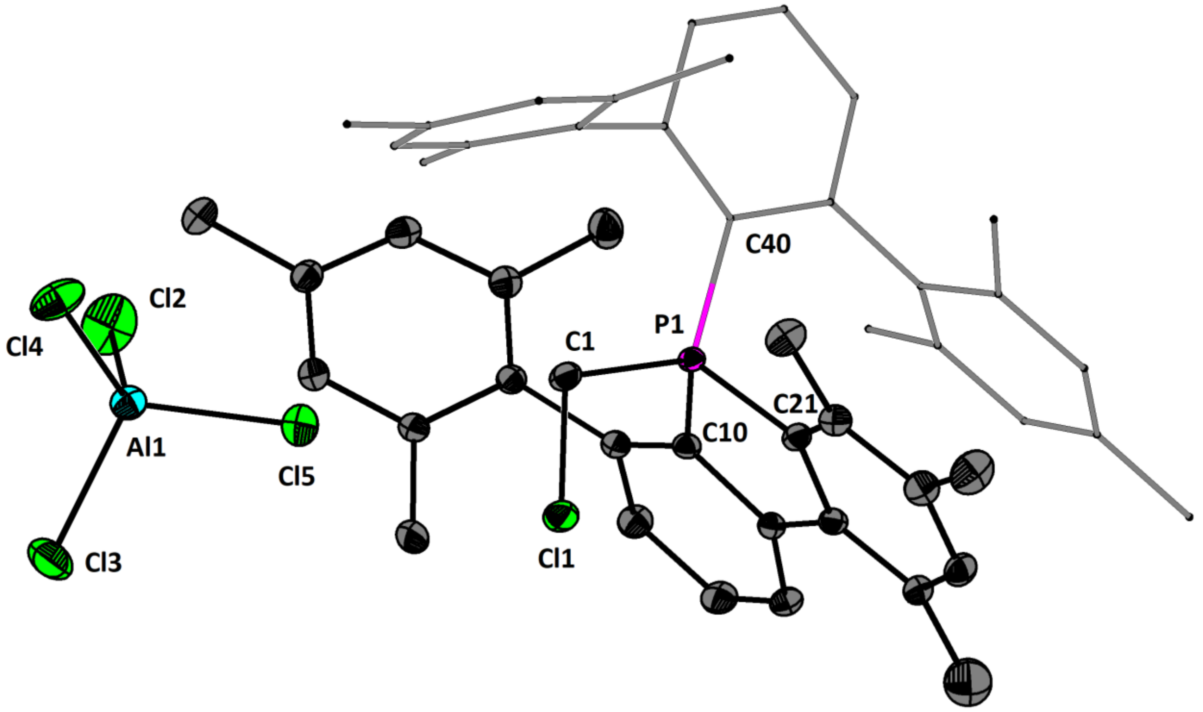

Figure S24. Molecular structures of [**4**][$AlCl_4$] showing 50% probability ellipsoids and the essential atom numbering scheme. Selected bond parameters [Å, °] of [**4**]$^+$: P1-C1 1.837(1), P1-C10 1.799(1), P1-C21 1.786(1), P1-C40 1.826(1), C10-P1-C40 113.14(5), C21-P1-C10 94.75(5), C21-P1-C40 114.04(5).

Table S1. Crystal data and structure refinement of (2,6-$Mes_2C_6H_3)_2AsF$, [**2a**][BAr^F_4] and **3a**.

	(2,6-$Mes_2C_6H_3)_2AsF$	[**2a**][BAr^F_4]	**3a**
Formula	$C_{48}H_{50}AsF$	$C_{80}H_{62}BF_{24}P$	$C_{48}H_{49}P$
Formula weight, g mol^{-1}	720.80	1521.07	656.84
Crystal system	Triclinic	Triclinic	Triclinic
Crystal size, mm	0.07×0.05×0.05	0.2×0.2×0.2	0.06×0.06×0.05
Space group	$P\bar{1}$	$P\bar{1}$	$P\bar{1}$
a, Å	10.957(5)	17.8152(7)	11.2904(5)
b, Å	11.542(5)	21.7642(8)	12.9577(6)
c, Å	16.417(5)	21.8095(8)	15.0899(7)
α, º	74.469(5)	68.0540(10)	72.905(2)
β, º	70.535(5)	76.9950(10)	84.767(2)
γ, º	78.956(5)	77.8320(10)	73.838(2)
V, Å^3	1874.0(13)	7566.9(5)	2026.57(16)
Z	2	4	2
ρ_{calcd}, g cm^{-3}	1.277	1.335	1.076
μ (Mo $K\alpha$), mm^{-1}	0.945	0.138	0.098
F(000)	760	3112	704
θ range, deg	4.74 to 61.2	4.654 to 56.752	4.62 to 51
Index ranges	$-15 \le h \le 15$	$-23 \le h \le 23$	$-13 \le h \le 12$
	$-16 \le k \le 16$	$-29 \le k \le 29$	$-15 \le k \le 15$
	$-23 \le l \le 23$	$-29 \le l \le 29$	$-18 \le l \le 18$
No. of reflns collected	248957	176027	40455
Completeness to θ_{max}	0.998	0.981	0.999
No. indep. Reflns	11512	37158	7558
No. obsd reflns with ($I>2\sigma(I)$)	10571	30061	6814
No. refined params	481	2053	454
GooF (F^2)	1.064	1.049	1.070
R_1 (F) ($I > 2\sigma(I)$)	0.0258	0.0705	0.0412
wR_2 (F^2) (all data)	0.0686	0.1702	0.1139
Largest diff peak/hole, e Å^{-3}	0.41/−0.48	0.98/−0.78	0.30/−0.32
CCDC number	1918428	1918429	1918430

Table S2. Crystal data and structure refinement of **3b** and [**4**][$AlCl_4$]

	**3b**	[**4**][$AlCl_4$]
Formula	$C_{48}H_{49}As$	$C_{49}H_{51}AlCl_5P$
Formula weight, g mol^{-1}	700.79	875.10
Crystal system	Triclinic	Triclinic
Crystal size, mm	0.3×0.2×0.2	0.08×0.06×0.05
Space group	$P\bar{1}$	$P\bar{1}$
a, Å	8.3779(3)	10.4702(4)
b, Å	13.7181(5)	14.6934(5)
c, Å	16.0602(5)	15.5911(5)
α, °	90.9720(10)	96.2860(10)
β, °	95.7560(10)	104.9190(10)
γ, °	101.3350(10)	105.3360(10)
V, Å^3	1799.38(11)	2194.23(13)
Z	2	2
ρ_{calcd}, g cm^{-3}	1799.38(11)	1.325
μ (Mo *Kα*), mm^{-1}	0.978	0.421
F(000)	740	916
θ range, deg	4.98 to 61.2	2.19 to 31.60
Index ranges	$-11 \le h \le 11$	$-15 \le h \le 15$
	$-19 \le k \le 19$	$-21 \le k \le 21$
	$-22 \le l \le 22$	$-22 \le l \le 22$
No. of reflns collected	43216	58416
Completeness to θ_{max}	0.996	0.997
No. indep. Reflns	10997	14694
No. obsd reflns with ($I>2\sigma(I)$)	9865	12524
No. refined params	454	517
GooF (F^2)	1.039	1.060
R_1 (*F*) ($I > 2\sigma(I)$)	0.0284	0.0377
wR_2 (F^2) (all data)	0.0697	0.1118
Largest diff peak/hole, e Å^{-3}	0.43/−0.31	0.88 / −0.73
CCDC number	1918431	1918432

Computational data

The structures of all starting materials, transition states, intermediates, and products were optimized in the gas-phase by density functional theory (DFT) at the B3PW91/6-311+G*[S11,S12] level of theory using Gaussian09.[S13] For the heavier atoms fully relativistic effective core potentials (Sb: ECP28MDF; Bi: ECP60MDF) and corresponding cc-pVTZ basis sets[S14,S15] were utilized. The starting geometries were modelled at the computer with GaussView 5. Transition states were calculated with the QST2 routine in Gaussian, using the optimized onset and end structures as starting point. Dispersion effects were accounted for using the empirical Grimme correction (GD3BJ).[S16]

References

S1. K. Ruhlandt-Senge, J. J. Ellison, R. J. Wehmschulte, F. Pauer, P. P. Power, *J. Am. Chem. Soc.* **1993**, *115*, 11353-11357.

S2. (a) N. A. Yakelis, R. G. Bergman, *Organometallics* **2005**, *24*, 3579–3581; (b) M. Brookhart, B. Grant, A. F. Volpe Jr., *Organometallics* **1992**, *11*, 3920-3922.

S3. M. Kuprat, M. Lehmann, A. Schulz and A. Villinger, *Organometallics*, **2010**, *29*, 1421-1427.

S4. M. Lehmann, A. Schulz, A. Villinger, *Angew. Chem., Int. Ed.* **2009**, *48*, 7444-7447.

S5. V. J. Scott, R. Çelenligil-Çetin, O. V. Ozerov, *J. Am. Chem. Soc.* **2005**, *127*, 2852-2853.

S6. J. B. Lambert, S. Zhang, C. L. Stern, J. C. Huffman, *Science* **1993**, *260*, 1917-1918.

S7. G. M. Sheldrick, *Acta Cryst*. **2008**, *A64*, 112-122.

S8. (a) L. Farrugia, *J. Appl. Cryst*. **1999**, *32*, 837-838. (b) O. V. Dolomanov, L. J. Bourhis, R. J. Gildea, J. A. K. Howard, H. Puschmann, *J. Appl. Cryst.* **2009**, *42*, 339-341.

S9. A. Spek *J. Appl. Cryst*. **2003**, *36*, 7-13.

S10. K. Brandenburg, Diamond, version 4.0.4, Crystal Impact GbR: Bonn, Germany, **2012**.

S11. J. P Perdew, J. A. Chevary, S. H. Vosko, K. A. Jackson, M. R. Pederson, D. J. Singh, C. Fiolhais, *Phys. Rev*. B **1992**, *46*, 6671-6687.

S12. A. D. Becke, *J. Chem. Phys*. **1993**, *98*, 5648-5652.

S13. M. J. Frisch, G. W. Trucks, H. B. Schlegel, G. E. Scuseria, M. A. Robb, J. R. Cheeseman, G. Scalmani, V. Barone, B. Mennucci, G. A. Petersson, H. Nakatsuji, M. Caricato, X. Li, H. P. Hratchian, A. F. Izmaylov, J. Bloino, G. Zheng, J. L. Sonnenberg, M. Hada, M. Ehara, K. Toyota, R. Fukuda, J. Hasegawa, M. Ishida, T. Nakajima, Y. Honda, O. Kitao, H. Nakai, T. Vreven, J. A. Montgomery, Jr., J. E. Peralta, F. Ogliaro, M. Bearpark, J. J. Heyd, E. Brothers, K. N. Kudin, V. N. Staroverov, R. Kobayashi, J. Normand, K. Raghavachari, A. Rendell, J. C. Burant, S. S. Iyengar, J. Tomasi, M. Cossi, N. Rega, J. M. Millam, M. Klene, J. E. Knox, J. B. Cross, V. Bakken, C. Adamo, J. Jaramillo, R. Gomperts, R. E. Stratmann, O. Yazyev, A. J. Austin, R. Cammi, C. Pomelli, J. W. Ochterski, R. L. Martin, K. Morokuma, V. G. Zakrzewski, G. A. Voth, P. Salvador, J. J. Dannenberg, S. Dapprich, A. D. Daniels, Ö. Farkas, J. B. Foresman, J. V. Ortiz, J. Cioslowski, D. J. Fox, Gaussian 09, Revision B.01, Gaussian Inc., Wallingford CT, **2010**.

S14. B. Metz, H. Stoll, M. Dolg, *J. Chem. Phys.* **2000**, *113*, 2563–2569.

S15. K. A. Peterson, *J. Chem. Phys.* **2003**, *119*, 11099–11123.

S16. S. Grimme, J. Antony, S. Ehrlich and H. Krieg, *J. Chem. Phys*., **2010**, *132*,154104.
